# Efficacy and Safety of Tangshen Formula on Patients with Type 2 Diabetic Kidney Disease: A Multicenter Double-Blinded Randomized Placebo-Controlled Trial

**DOI:** 10.1371/journal.pone.0126027

**Published:** 2015-05-04

**Authors:** Ping Li, Yiping Chen, Jianping Liu, Jing Hong, Yueyi Deng, Fang Yang, Xiuping Jin, Jing Gao, Jing Li, Hui Fang, Geling Liu, Liping Shi, Jinhang Du, Yang Li, Meihua Yan, Yumin Wen, Wenying Yang

**Affiliations:** 1 Institute of Clinical Medical Science, China-Japan Friendship Hospital, Beijing, China; 2 Department of Nephrology, Longhua Hospital, Shanghai University of TCM, Shanghai, China; 3 Centre for Evidence-Based Chinese Medicine, Beijing University of Chinese Medicine, Beijing, China; 4 Department of Endocrinology, China-Japan Friendship Hospital, Beijing, China; 5 Research Center of Experimental Medicine, Hebei United University, Tangshan, China; 6 Department of Endocrinology, Hebei United University Affiliated Hospital, Tangshan, China; 7 Department of Nephrology, Dongzhimen Hospital Affiliated to Beijing University of Chinese Medicine, Beijing, China; 8 Department of Endocrinology, Tangshan Gongren Hospital, Tangshan, China; 9 Department of Endocrinology, Kailuan General Hospital, Tangshan, China; 10 Department of Nephrology, China-Japan Friendship Hospital, Beijing, China; 11 School of Statistics, Renmin University of China, Beijing, China; Pennington Biomedical Research Center, UNITED STATES

## Abstract

**Background:**

Persons with diabetes are at high risk of developing diabetic kidney disease (DKD), which is associated with high morbidity and mortality. Current drug therapies for DKD, such as angiotensin-converting enzyme inhibitors (ACEIs) and angiotensin receptor blockers (ARBs), are not entirely satisfactory. This study aimed to evaluate the additional benefit and safety of the Chinese herbal granule Tangshen Formula (TSF) in treating DKD.

**Methods:**

The study was designed as a six-center randomized, double-blind, placebo-controlled trial. From April 2007 through December 2009, 180 patients with DKD were enrolled. In addition to conventional treatment with ACEIs or ARBs, 122 participants were randomly assigned to receive TSF and 58 participants to receive placebo for 24 weeks. Primary outcome was urinary protein level, measured by urinary albumin excretion rate (UAER) for participants with microalbuminuria, 24-hour urinary protein (24h UP) for participants with macroalbuminuria. Secondary outcomes included renal function, serum lipids, quality of life, symptoms, and adverse events.

**Findings:**

After 24 weeks of treatment, no statistically significant difference in UAER (TSF −19.53 μg/min compared with placebo −7.01 μg/min, with a mean difference of −12.52 μg/min; 95%CI, −68.67 to 43.63, *P* = 0.696) was found between TSF and placebo groups. However, TSF displayed a statistically significant decrease in 24h UP (TSF−0.21 g compared with placebo 0.36 g, with a mean difference of −0.57g; 95%CI, −1.05 to −0.09, *P* = 0.024). Estimated glomerular filtration rate (eGFR) was improved in both patients with microalbuminuria and macroalbuminuria, with a mean difference of 15.51 ml/min/1.73 m^2^ (95%CI, 3.71 to 27.31), 9.01 ml/min/1.73 m^2^ (95%CI, −0.10 to 18.13), respectively. Other secondary outcomes showed no statistically significant difference between groups or in the incidence of adverse events.

**Conclusions:**

Based on conventional treatments, TSF appears to provide additional benefits compared with placebo in decreasing proteinuria and improving eGFR in DKD patients with macroalbuminuria. Nevertheless, further study is needed to evaluate TSF treating patients with microalbuminuria.

**Trial Registration:**

Chinese Clinical Trial Registry ChiCTR-TRC-10000843

## Introduction

Diabetic kidney disease (DKD) is a common complication of diabetes mellitus (DM). It is characterized by albuminuria and loss of kidney function [1]. DKD is also the leading cause of end-stage renal diseases (ESRD) in developed countries [2]. As the prevalence of DM increases worldwide [3,4], there has been a concomitant increase in the incidence of DKD. In China, a large study has projected that there are 113.9 million persons affected by diabetes [5]. Extrapolating from cross-sectional studies that have found micro- or macroalbuminuria affects up to 60% of Asian patients [6], it is possible that some 68 million persons with diabetes in China may have DKD. This staggering number places a tremendous burden on the healthcare system.

At present, recommended therapies for DKD include renin-angiotensin-aldosterone system blockade, antihypertensive drugs, glycemic control, and antilipemic agents [7]. Angiotensin-converting enzyme inhibitors (ACEIs) and angiotensin receptor blockers (ARBs) have been documented to delay the progression of DKD by preventing generation of albuminuria, reducing microalbuminuria level, and slowing deterioration of renal function [8,9]. However, these positive effects are mitigated by negative findings. For example, in the Reduction of Endpoints in NIDDM with the Angiotensin II Antagonist Losartan (RENAAL) study with patients of type 2 diabetes and nephropathy, 43.5% of participants in the losartan (ARB) group as compared with 47.1% in the placebo arm experienced doubling of serum creatinine concentration, ESRD, or death [10]. Thus, losartan did not have a significant impact on these primary outcomes. Moreover, the main side effects of ACEIs and ARBs, such as dry cough, rise in serum potassium and rise in serum creatinine, limit their application, especially in patients with glomerular filtration rate (GFR) <60ml/min/1.73 m^2^. In the past decade, several clinical trials investigating new medications for DKD have been undertaken, but most medications failed or trials were terminated due to either poor efficacy or serious adverse events [11,12].Therefore, more effective treatments for DKD need to be explored.

Traditional Chinese medicine (TCM) is a medical practice based on syndrome differentiation. Chinese herbal medicine (CHM) is the main therapeutic modality of TCM that uses a combination of plants, minerals and animal parts for maintenance of health and treatment of diseases. Records of using CHM to treat diabetes and kidney disease can be found in the ancient TCM literature. In modern times, CHM is being applied as either a primary or complementary therapy for kidney disease in China. Multicenter randomized controlled clinical trials have shown that CHM therapy improves estimated glomerular filtration rate (eGFR) in both patients with stage 3chronic kidney disease (CKD) and idiopathic membranous nephropathy [13,14]. Many CHM preparations have been used in treating CKD in China, and systematic reviews have shown that some are effective [15,16]. Investigations on CHM treatment of DKD have been undertaken in China. Results indicate that CHM may provide a greater benefit in reducing urinary protein level compared with either placebo control group or ACEI/ARB control group [17].However, most of the clinical trials with these CHM preparations were either of small sample size, not well-randomized, or absent of quality control. Thus, well-designed, multicenter randomized controlled clinical trials with large sample sizes are required to evaluate efficacy and safety of CHM treatment for DKD.

Tangshen Formula (TSF) is a CHM remedy for DKD based on empirical evidence gleaned from Chinese medicine practitioners. Its efficacy has been explored in experimental laboratory and clinical observation studies [18–20].In this study, a prospective, multicenter, double-blind, randomized controlled study was undertaken to evaluate the benefit and safety of TSF for treatment of early stage DKD when used with ACEIs or ARBs.

## Methods

### Ethics statement

The protocol for this trial and supporting CONSORT checklist are available as supporting information; see S1 CONSORT Checklist and S1 and S2 Protocols.This study was designed as a six-center, randomized, double-blind, placebo-controlled clinical trial. The protocol (No. 2006–059) was approved by the ethics committee of the China-Japan Friendship Hospital, which oversaw the study. The protocol was registered with the Chinese Clinical Trial Registry (ChiCTR-TRC-10000843). The study was conducted in accordance with the principles of the Declaration of Helsinki (2004 version). All included patients signed written informed consent documents.

This study was registered after patient recruitment began but before completion of data analysis as the funding agency did not require registration of clinical trials. The authors certify that all ongoing and related trials for this drug/intervention have been registered.

### Setting and Participants

Inpatients and outpatients with DKD were recruited from April 2007 through December 2009 among departments of endocrinology and nephrology of six hospitals in China: China-Japan Friendship Hospital, Beijing; Longhua Hospital affiliated to Shanghai University of TCM, Shanghai; Dongzhimen Hospital affiliated to Beijing University of Chinese Medicine,Beijing; Hebei United University School of Medicine Affiliated Hospital, Tangshan; Kailuan General Hospital, Tangshan; and Tangshan Gongren Hospital, Tangshan.

### TCM syndrome differentiation

According to clinical research guidelines for new investigational drugs in traditional Chinese medicine [21] and characteristics of DKD, the diagnostic standards of deficiency of both Qi and Yin with blood stasis syndrome were as follows: (1) Primary symptoms and signs include fatigue, weakness and soreness of the low back and knees, heat sensation in the palms and soles, dry mouth and throat, and listlessness. (2)Secondary symptoms and signs include catching cold easily, pale complexion, irritability, numbness, edema, frequent urination at night, constipation, and hematuria. Participants who exhibited no less than two of the primary symptoms and at least two of the secondary symptoms were diagnosed as deficiency of both Qi and Yin with blood stasis syndrome.

Four grades with different values were assigned to each symptom and the values for primary symptoms were two times greater than secondary symptoms. Therefore, each primary symptom sign was scored as 0, 2, 4, or 6, while a secondary symptom or sign was scored as 0, 1, 2, or 3.The total score of the participant was designated as the TCM symptom score.

### Inclusion Criteria and Exclusion Criteria

#### Inclusion criteria

Type 2 diabetes was defined by American Diabetes Association guidelines (ADA; 2006) [22]. Diabetic kidney disease was defined based on diagnostic criteria of the National Kidney Foundation Kidney Disease Outcomes Quality Initiative (NKF-KDOQI; 2007) [23].All participants had a urinary albumin excretion rate (UAER) >20 μg/min, and/or 24-hour urinary protein (24h UP) between 0.5 and 2.0 g/d, and eGFR estimated by Cockcroft-Grault equation between 60 ml/min and 130 ml/min. Other inclusion criteria were BP<140/90 mmHg, fasting blood glucose (FBG) ≤7.8 mmol/L and A1C ≤7.5%. TCM syndrome of all patients was deficiency of both Qi and Yin with blood stasis. Participants ranged in age from 25 to 75.

#### Exclusion criteria

Patients with the following conditions were excluded from the trial: history of primary kidney disease or systemic disease with elevated urinary protein; history of other endocrine and/or metabolic disease; history of myocardial infarction, angina pectoris, or other recent cardiovascular problem (including cerebrovascular event) within 3 months prior to signing informed consent; impaired hepatic function with alanine transaminase (ALT) and/or aspartate aminotransferase (AST) of 2-fold the upper limit of normal level or above; fasting serum triglyceride >10mmol/L (>886 mg/dl); herbal allergy; recent infection within 4 weeks; pregnancy or lactating; mental disorder or non-cooperation; use of glucocorticosteroids, thiazide diuretics, or niacin within the last 3 months.

### Interventions

After initial screening, all participants entered a 2-week run-in period with diet control and programmed daily exercise. According to ADA recommendations, all participants received either an ACEI or ARB agent [22]. Antihypertensive treatment, glycemic control, and antilipemic agents were adopted as conventional treatments using open-label drugs (calcium channel blockers, insulin, statins). Subsequently, eligible patients were randomly assigned to receive either 8 grams placebo or 8 grams TSF granule dissolved in warm water taken orally, twice daily. The intervention period was 24 weeks.

#### Preparation of TSF and placebo

Both TSF (Lot number0606320) and the placebo were prepared and standardized by an established company recognized for high quality control standards: Jiangyin Tianjiang Pharmaceutical, Jiangsu, China (http://www.tianjiang.com). TSF consists of seven natural herbs: astragalus (*A*.*membranaceus* (Fisch.) Bge.), burning bush (*E*.*alatus* (Thunb.) Sieb.), rehmannia (*R*.*glutinosa* Libosch), bitter orange (*C*.*aurantium* L.), cornus (*C*.*officinalis* Sieb. Et Zuce), rhubarb (*R*.*palmatum* L.) and notoginseng (*P*.*notoginseng* (Burk.) F.H. Chen) (Table 1). Each component in TSF was produced by soaking in distilled water for 30 minutes, boiling in 10 volumes of water (v/w) for 1 hour, extracting with water twice, filtrating and condensing to the concentration of 1 g/ml and processed to fine granular by spray drying. The final product was made by combining the individual herbal granules in the proportions indicated in Table 1.TSF is a dispensing formula in its scientific research stage and has not yet been licensed for clinical use in China.

**Table 1:** Composition of Tangshen Formula.

Common English Name	Pharmaceutical Name	Latin Botanical Name	Powdered Herb (%)
Astragalus root	Astragali Radix	*Astragalus membranaceus* (Fisch.) Bge.	35.3
Burning bush twig	Euonymi Ramulus	*Euonymus alatus*(Thunb.) Sieb.	17.6
Rehmannia root	Rehmanniae Radix	*Rehmannia glutinosa* Libosch	14.1
Bitter orange	Aurantii Fructus	*Citrus aurantium* L.	11.8
Cornus fruit	Corni Fructus	*Cornus officinalis* Sieb. et Zuce	10.6
Rhubarb root and rhizome	Rhei Radix et Rhizoma	*Rheum palmatum* L.	7.1
Notoginseng root	Notoginseng Radix	*Panax notoginseng* (Burk.) F.H. Chen	3.5

Ingredients of the placebo were lactose (78.43%), maltodextrin (14.88%), tartrazine (0.07%), sunset yellow (0.026%), caramel (6.5%), picric acid (0.026%), and sucralose (0.07%).These were prepared by the same company as TSF. Based on our knowledge, none of the ingredients in the placebo at this dosage has been reported to have physiological effects.

#### Chemical analysis of TSF

Quality control of raw herbs and final granule product was performed according to the *Chinese Pharmacopoeia* [24]. Chemical composition of TSF was validated using high-performance liquid chromatography/mass spectrometry (HPLC/MS). Nine most representative compounds were identified in TSF, which were used as the quality control markers for TSF.

### Outcome Measures

Participants were followed up by their physicians once every 4 weeks. Primary outcome measured was urinary protein level, assessed by UAER for patients with microalbuminuria, 24h UP for patients with macroalbuminuria. Secondary outcomes were renal functions including: eGFR, serum creatinine, blood urea nitrogen; lipid profiles including: total cholesterol (TC), triglyceride (TG), low density lipoprotein (LDL), high density lipoprotein (HDL); and quality of life using the mainland Chinese version of the World Health Organization Quality of Life questionnaire (WHOQOL-BREF) [25] and the Diabetes Quality of Life survey (DQOL) [26]. In addition, TCM symptom scores were assessed [21]. Routine blood and urine tests, electrocardiogram, ALT and AST were performed as safety indicators. All outcomes were evaluated at baseline, 12 weeks and 24 weeks.

### Randomization and Blinding

SPSS 10 software (Softonic International, Barcelona, Spain) was used to generate random allocation sequence based on blocked randomization with a block size of six. The randomization list was maintained by an independent clinical research coordinator at the World Federation of Chinese Medicine Societies, Beijing. At the suggestion of the ethics committee, participant ratio in the TSF group and the placebo group was set at 2:1. Investigators of each center enrolled participants sequentially according to their consultation order. Each participant was assigned a unique number, which was used throughout the trial.

Double-blinding was adopted in this study. All individuals including participants, physicians, statistician and outcome assessors were unaware of the random sequence and drug assignments except of the person overseeing drug assignment, who was not involved in the study and was responsible for supervision, instructing participants on how to take the drugs and compliance recording of the drugs. Both TSF and placebo granules were similar in packaging, appearance, shape, size and color. The project department of World Federation of Chinese Medicine Societies examined the data in blinded fashion, except of when serious adverse events occurred for which a causal relationship with the study drug cannot be ruled out.

### Statistical Methods

Sample size was estimated according to preliminary results [18]. Mean reduction of UAER in the conventional treatment group was 30.19μg/min; mean reduction of UAER in TSF group was 81.67 μg/min, resulting an effect size of 51.48μg/min with 90% power to detect and α of 0.05. Sample size was estimated to be 56 in the placebo group, and 112 in the TSF group. Assuming a dropout rate of 15%, sample size was estimated to be 192. A database was built via double entry into Epidata 3.0 (The EpiData Association, Odense, Denmark). The intention-to-treat (ITT) population, which included participants having baseline data and at least one post-treatment assessment, was used to conduct efficacy analyses. All randomly assigned participants were covered for safety analyses. Due to different measurement methods of urinary protein in microalbuminuria and macroalbuminuria stages, comparisons were calculated between the treatment and placebo group in each DKD stage separately. The mean and standard deviation were calculated for continuous variable. The frequency and percentage were used for the numerical data. For measurement of the primary and secondary outcomes, we compared the change within each treatment group from baseline to week 24 (end point), and compared the change between each treatment group. Data normalcy were assessed by Shapiro's test. For normally distributed data, analyses used paired t-test for paired samples and t-test for independent samples. For data that were not normally distributed, we used the nonparametric method of Wilcoxon rank-sum test for comparison between groups. 95% confidence intervals around the means for each comparison group were also calculated. For the safety evaluation, a chi-square test was used to compare the incidence of adverse events between the two groups. A *P*-value less than 0.05 was considered significant. SAS9.2 software (SAS Institute Inc., Cary, NC, USA) was used for analyses.

## Results

### Enrollment and Study Population

**Fig 1:**
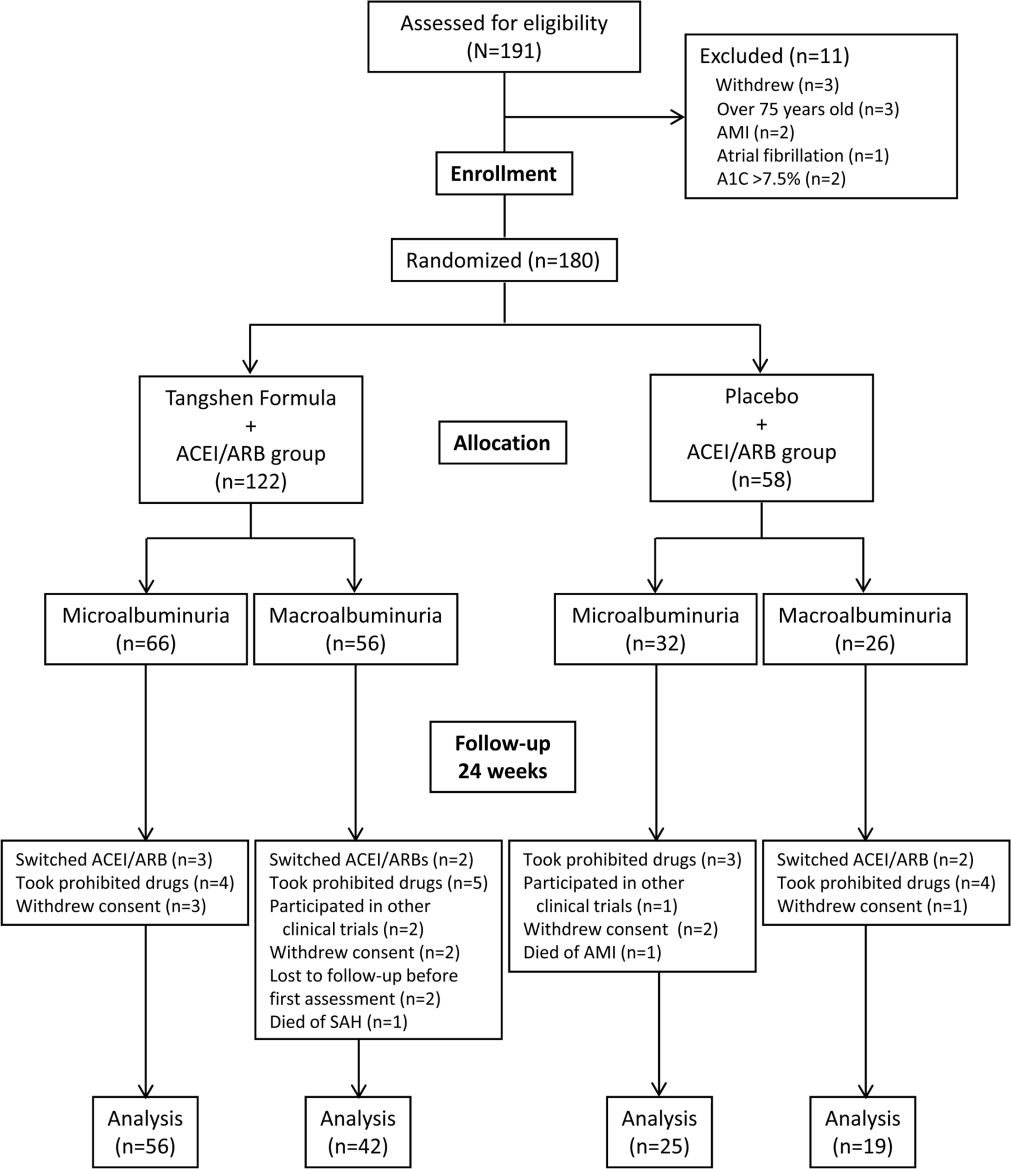
Screening, randomization, and completion of evaluations.

A total of 191 patients from six medical centers in China were screened from April 2007 through December 2009. Of the 191 patients, three withdrew consent; three were over 75 years old; two had a history of AMI and received coronary stenting within 3 months prior to signing informed consent. One patient had atrial fibrillation and was on an anticoagulant; two had an A1C above 7.5%. The final enrollment was 180 participants, of which 98 had microalbuminuria and 82 had macroalbuminuria; 122 participants were randomly assigned to receive TSF and 58 participants to receive placebo. During the intervention period, protocol violation occurred in 26 participants, including 7 who switched ACEI or ARB agents due to uncontrolled high blood pressure, 16 who took prohibited drugs such as other TCM remedies or patent medicines, or niacin, and 3 who participated in other clinical trials. Eight participants withdrew consent. Two were lost to follow-up after first assessment. Two participants died. After 24 weeks, 81 participants with microalbuminuria remained with 56 participants in the TSF group and 25 in the placebo group; 61 participants with macroalbuminuria remained with 42 in the TSF and 19 in the placebo group (Fig 1 1). The groups were well-balanced with regard to baseline clinical and demographic characteristics (Table 2).

**Table 2:** Baseline characteristics of DKD patients.

	Microalbuminuria			Macroalbuminuria		
	TSF(n = 66)	Placebo (n = 32)	*P*	TSF (n = 56)	Placebo (n = 26)	*P*
**Age** (yr) ^a^	59.48±10.059	56.72±9.38	0.195 ^b^	58.88±8.96	60.81±9.91	0.402 ^b^
**Male/Female**	36/30	17/15	0.895 ^c^	33/23	14/12	0.665 ^c^
**BMI** (kg/m^2^) ^a^	25.02±3.31	25.68±3.43	0.677 ^c^	25.98±3.54	25.76±2.85	0.765 ^c^
**Blood pressure**						
Systolic (mmHg) ^a^	127.57±9.01	126.44±8.18	0.501 ^c^	130.02±14.1	130.19±7.28	0.72 ^c^
Diastolic (mmHg) ^a^	77.49±7.38	78.19±6.77	0.59 ^c^	78.61±7.7	79.31±8.05	0.628 ^c^
**Medical history**						
Diabetes (yr) ^a^	9.67±6.05	8.06±6.2	0.129 ^c^	11.88±6.95	1±7.41	0.408 ^c^
Hypertension (%)	41 (62.1)	21 (65.6)	0.736 ^c^	39 (69.6)	17 (65.4)	0.7 ^c^
CVD (%)	12 (18.2)	5 (15.6)	0.754 ^c^	11 (19.6)	5 (19.2)	0.965 ^c^
Stroke (%)	8 (12.1)	3 (9.4)	0.686 ^c^	2 (3.6)	1 (3.8)	0.951 ^c^
TCM score ^a^	12.8±7.16	10.34±7.97	0.069 ^c^	13.89±7.96	12.73±6.81	0.499 ^c^
**Laboratory variables** ^a^						
UAER (μg/min)	105.39±77.29	107.21±72.4	0.889 ^c^	—	—	—
24h UP (g)	—	—	—	1.12±0.75	0.84±0.64	0.188 ^c^
eGFR(ml/min)	89.44±29.77	107.12±50	0.2 ^c^	86.2±32.59	81.39±31.90	0.622 ^c^
SCr(μmol/ L)	73.4±18.8	71.58±20.55	0.73 ^c^	85.57±27.23	94.38±43.07	0.821 ^c^
BUN (mmol/L)	5.91±1.91	6.03±1.95	0.78 ^c^	5.93±1.84	6.07±1.90	0.943 ^c^
TC (mmol/L)	5.11±1.30	5.20±1.71	0.786 ^c^	5.27±1.78	5.39±1.52	0.746 ^c^
TG (mmol/L)	1.81±1. 15	1.99±1. 49	0.796 ^c^	2.16±1.38	2.01±1.03	0.940 ^c^
HDL(mmol/L)	1.24±0.32	1.27±0.41	0.81 ^c^	1.27±0.45	1.34±0. 37	0.187 ^c^
LDL (mmol/L)	3.23±1.02	3.17±1.04	0.789 ^b^	3.08±0.99	3.274±1.32	0.511 ^b^
A1C (%)	6.92±1.27	6.88±1.04	0.87 ^c^	6.94±1.11	7.56±2.61	0.38 ^c^

Abbreviations: A1C, hemoglobin A1c; BMI, body mass index; BP, blood pressure; BUN, urea nitrogen; CVD, cardiovascular disease; eGFR, estimated glomerular filtration rate; HDL, high-density lipoprotein; LDL, low-density lipoprotein; SCr, serum creatinine; TC, total cholesterol; TCM, traditional Chinese medicine; TG, triglycerides; UAER, urinary albumin excretion rate; UP, urinary protein.

^a^Data are presented as mean ± SD.

^b^Students t-test.

^c^Wilcoxon Rank-sum test.

### Primary Outcomes

#### Urinary albumin excretion rate (UAER)

For participants with microalbuminuria, baseline values of UAER in the TSF group (105.39±77.29 μg/min) and placebo group (107.21 ± 72.4 μg/min) were similar (Table 2). After 24 weeks of treatment, UAER was 88.37 ± 108.46 μg/min in the TSF group and114.9 ± 98.25 μg/min in the placebo group. Changes in UAER between baseline and 24 weeks treatment were −19.53 μg/min (95%CI, −52.47 to 13.41, *P*  = 0 .021) in the TSF group and −7.01 μg/min (95%CI, −47.33 to 33.73 *P*  =  0.445) in the placebo group. Mean difference in change of UAER between the two groups was not statistically significant (−12.52 μg/min, 95%CI, −68.67 to 43.63, *P*  =  0.696) (Table 3).

**Table 3:** Effect of TSF and placebo on the primary and secondary outcomes in DKD patients with microalbuminuria.

	TSF			Placebo			TSFvs. Placebo
Variable	Baseline ^a^(n = 66)	24 weeks ^a^ (n = 56)	Change (95%CI)	Baseline ^a^ (n = 32)	24 weeks ^a^ (n = 25)	Change (95%CI)	Estimate (95%CI)
**UAER (μg/min)**	105.39±77.29	88.37± 108.46	−19.53 ^b^(−52.47,13.41)	107.21±72.4	114.9 ± 98.25	−7.01(−47.33,33.73)	−12.52(−68.67,43.63)
**eGFR (ml/min)**	89.44±29.77	94.80± 33.76	5.89(−0.43,12.21)	107.12±50	105.34± 43.71	−9.62(−20.70,1.46)	15.51(3.71,27.31)
**SCr (μmol/ L)**	73.4±18.8	68.25± 17.95	-4.87(−8.51, −1.23)	71.58±20.55	71.98± 23.30	5.06(−4.11,14.23)	−9.93(−17.92, −1.94)
**BUN (mmol/L)**	5.91±1.91	5.98± 1.59	0.24(−0.20,0.68)	6.03±1.95	5.84± 1.78	−0.46(−1.23,0.31)	0.70(−0.13,1.53)
**TC (mmol/L)**	5.11±1.30	5.21±1.27	0.02(−0.32,0.36)	5.20±1.71	5.51±1.97	0.32(0.46,1.11)	−0.31(−1.15,0.54)
**TG (mmol/L)**	1.81±1.15	1.88±1.22	0.16(−0.18,0.51)	1.99±1. 49	2.14±1.24	0.05(−0.62, −0.72)	0.12(−0.55,0.79)
**HDL(mmol/L)**	1.24±0.32	1.27±0.31	0(−0.01,0.07)	1.27±0.41	1.21±0.25	−0.06(−0.26,0.14)	0.05(−0.16,0.26)
**LDL (mmol/L)**	3.23±1.02	3.18±0.95	-0.23 (−0.47,0.02)	3.17±1.04	3.37±1.84	0.2(−0.42,0.82)	−0.43(−1.09,0.23)
**A1C (%)**	6.92±1.27	6.88±1.11	−0.04(−0.33,0.25)	6.88±1.04	7.02±1.34	0.03(−0.56,0.62)	−0.07(−0.65,0.51)
**Systolic (mmHg)**	127.57±9.01	126.68±10.22	−1.57(−3.78,0.64)	126.44±8.18	126.96±6.07	0.72(−3.09,4.53)	−2.29(−6.39,1.80)
**Diastolic (mmHg)**	77.49±7.381	76.64±7.7	−1.07(−3.12,0.98)	78.19±6.77	76.52±6.7	−1.76(−4.63,1.11)	0.69(−2.88,4.25)

Abbreviations: A1C, hemoglobin A1c; BP, blood pressure; BUN, urea nitrogen; eGFR, estimated glomerular filtration rate; HDL, high-density lipoprotein; LDL, low-density lipoprotein; SCr, serum creatinine TC, triglycerides; TG, total cholesterol; UAER, urinary albumin excretion rate.

^a^Data presented as mean±SD.

^b^ *P* = 0.021

#### 24-hour urinary protein (24h UP)

For participants with macroalbuminuria, 24h UP was employed. Baseline values of 24h UP in the TSF group (1.12 ± 0.75 g) and placebo group (0.84 ± 0.64 g) were similar (Table 2). After 24 weeks of treatment, 24h UP was 0.91 ± 0.90 g in the TSF group and 1.20 ± 1.10 g in the placebo group. Changes in urinary protein excretion between baseline and 24 weeks treatment were −0.21 g (95%CI, −0.48 to 0.06, *P* = 0.017) in the TSF group and 0.36 g (95%CI, −0.04 to 0.76, *P* = 0.134) in the placebo group. Mean difference in change in 24h UP between the two groups was statistically significant (−0.57 g, 95%CI, −1.05 to −0.09, *P* = 0.024) (Table 4).

**Table 4:** Effect estimation for primary and secondary outcomes in DKD patients with macroalbuminuria.

	TSF			Placebo			TSFvs. Placebo
Variable	Baseline ^a^ (n = 56)	24 weeks ^a^ (n = 42)	Change (95%CI)	Baseline ^a^ (n = 26)	24 weeks ^a^ (n = 19)	Change (95%CI)	Estimate(95%CI)
**24h UP (g)**	1.12±0.75	0.91±0.90	−0.21 ^b^ (−0.48,0.06)	0.84±0.64	1.20±1.10	0.36(−0.04,076)	−0.57 ^c^(−1.05, −0.09)
**eGFR (ml/min)**	86.2±32.59	90.34±44.38	1.96 (−5.26,9.18)	81.39±31.90	75.63±23.25	−7.05(−12.98, −1.12)	9.01(−0.10,18.13)
**SCr (μmol/L)**	85.57±27.23	87.27±33.22	3.91(−2.98, 10.79)	94.38±43.07	93.77±34.51	9.14 (2.10,16.18)	−5.24(−16.18,5.70)
**BUN (mmol/L)**	5.93±1.84	7.81±3.54	0.77 (−0.04,1.58)	6.07±1.90	7.37±2.81	0.78(0.05,1.51)	−0.01(−1.07,1.06)
**TC (mmol/L)**	5.27±1.78	5.21±1.26	0.42 (−0.09,0.93)	5.39±1.52	5.52±1.34	−0.16 (−0.83,0.52)	0.57(−0.25,1.40)
**TG (mmol/L)**	2.16±1.38	1.74±0.80	−0.42 (−0.93,0.10)	2.01±1.03	1.76±1.29	−0.24 (−1.03,0.54)	−0.18(−1.06,0.70)
**HDL (mmol/L)**	1.27±0.45	1.31±0.41	0.00 (−0.11,0.10)	1.34±0. 37	1.33±0.36	0.02 (−0.11,0.15)	−0.03(−0.19,0.14)
**LDL (mmol/L)**	3.08±0.99	2.91±0.76	−0.13(−0.49, 0.24)	3.27±1.32	3.24±0.98	−0.08 (−0.66,0.50)	−0.05(−0.68,0.59)
**A1C (%)**	6.94±1.11	7.11±1.44	0.14 (−0.20,0.47)	7.56±2.61	6.87±0.68	−0.76 (−2.08,0.56)	0.89(−0.46,2.25)
**Systolic (mmHg)**	130.02±14.1	128.66±12.01	−1.41 (−0.63,3.56)	130.19±7.28	127.94±8.71	−3.17(−5.77, −0.57)	1.75(−3.77,7.27)
**Diastolic (mmHg)**	78.61±7.7	78.17±7.12	0.24 (−2.39,2.88)	79.31±8.05	80.22±6.49	−0.44(−3.43,2.54)	0.69(−3.69,5.06)

Abbreviations: A1C, hemoglobin A1c; BP, blood pressure; BUN, urea nitrogen; eGFR, estimated glomerular filtration rate; HDL, high-density lipoprotein; LDL, low-density lipoprotein; SCr, serum creatinine; TC, triglycerides; TG, total cholesterol; UP, urinary protein;

^a^Data presented as mean±SD.

^b^ *P* = 0.017.

^c^ *P* = 0.024.

### Secondary Outcomes

#### Estimated glomerular filtration rate (eGFR)

For patients with microalbuminuria, baseline values of eGFR in the TSF group (89.44 ± 29.77 ml/min/1.73 m^2^) and the placebo group (107.12 ± 50 ml/min/1.73 m^2^) were similar (Table 2). After 24 weeks of treatment, eGFR was 94.80±33.76ml/min/1.73m^2^ in the TSF group and 105.34±43.71ml/min/1.73m^2^ in the placebo group. Changes were 5.89ml/min/1.73 m^2^ (95%CI, −0.43 to 12.21) in the TSF group and −9.62ml/min/1.73 m^2^ (95%CI, −20.70 to 1.46) in the placebo group. Mean difference in change of eGFR between the two groups was 15.51ml/min/1.73 m^2^ (95%CI, 3.71 to 27.31) (Table 3). For patients with macroalbuminuria, baseline values of eGFR in the TSF group (86.2 ± 32.59ml/min/1.73 m^2^) and the placebo group (81.39 ± 31.90ml/min/1.73 m^2^) were similar (Table 2). After 24 weeks of treatment, eGFR was 90.34 ± 44.38ml/min/1.73 m^2^ in the TSF group and 75.63 ± 23.25ml/min/1.73 m^2^ in the placebo group. Changes were 1.96ml/min/1.73 m^2^ (95%CI, −5.26 to 9.18) in the TSF group and −7.05 ml/min/1.73 m^2^ (95%CI, −12.9 to −1.12) in the placebo group. Mean difference in change of eGFR between the two groups was 9.01 ml/min/1.73 m^2^ (95%CI, −0.10 to 18.13) (Table 4).

#### TCM symptom scores

Baseline TCM symptom scores were 13.44 ± 7.7 in the TSF group and 11.54 ± 7.71 in placebo group. After12 weeks of treatment, TCM scores declined to 9.29 ± 6.37 in the TSF group and to 8.47 ± 6.01 in the placebo group. After 24 weeks of treatment, TCM symptom scores were 7.76 ± 5.29 in the TSF group and 7.52 ± 6.33 in the placebo group.

Changes in scores at the end of weeks 12 and 24 were examined using linear mixed-effects model. There was a significant time-group interaction effect, as scores in the TSF group declined more than those in the placebo group in week 24 (*P* = 0.0371).

### Other Secondary Outcomes

Although there was a remarkable change in LDL in participants with microalbuminuria after treatment of TSF, no statistically significant difference in other blood lipid levels (TG, TC, HDL), A1C, and BP between the TSF and placebo groups were detected (Tables 3 and 4). Moreover, scores in all domains and overall were not significantly different between groups in either WHOQOL-BREF or DQOL.

### Adverse Events

Of the 180 total participants, 17 adverse events were reported (Table 5). Eight of these events were in the placebo group and nine events in the TSF group (*P* = 0.169). Two participants died during the research period: 1 person of subarachnoid hemorrhage (SAH) in the TSF group (1/122, 0.82%) and 1 person of acute myocardial infarction (AMI) in the placebo group (1/56, 1.72%). Elevated liver enzyme (ALT/AST <2-fold the upper limit of normal) occurred in 5 participants (5/122, 5.1%) in the TSF group as compared with 4 participants (4/58, 9.09%) in the placebo group (*P* = 0.47). Of these 9 participants, 5 (4 cases in the TSF group and 1 in the placebo group) had ALT/AST elevated at week 12, but at week 24 ALT/AST returned to normal without liver-protecting treatment. One participant in the TSF group had a urinary tract infection at week 12 (1/122, 0.82%), and 1 participant in the placebo group developed pneumonia at week 12 (1/56, 1.72%), with both recovering following antibiotic treatment. Two participants in the TSF group had mild anemia (2/122, 1.94%), and 1 participant in the placebo group had moderate anemia (1/56, 1.72%).

**Table 5:** Adverse events in TSF and placebo groups expressed as number of events.

	TSF(n = 122)	Placebo(n = 58)
**Elevated ALT/ AST** ^**a**^	5 (5.1%)	4 (9.09%)
**Acute myocardial infarction**	0	1 (1.72%)
**Death**	1 (0.82%)	1 (1.72%)
**Infection**	1 (0.82%)	1 (1.72%)
**Anemia**	2 (1.94%)	1 (1.72%)
**Total**	9	8

Abbreviations: ALT, alanine transaminase; AST, aspartate aminotransferase

^a^ 2-fold or higher than upper limit of normal.

## Discussion

This study shows that after 24 weeks of intervention, TSF along with ACEI/ARB treatment significantly reduced 24h UP and promoted GFR in DKD patients with macroalbuminuria. Because progression of albuminuria is a surrogate outcome for progression of DKD, we deduce that TSF may delay the progression of DKD to end-stage renal failure in DKD patients with macroalbuminuria. In the current study, TSF had a positive effect on macroalbuminuria levels, but did not have significant impact on microalbuminuria levels. There are several possible reasons for this phenomenon. One main reason could be that ACEIs or ARBs were used in both groups as a conventional treatment and these drugs are well known to reduce microalbuminuria in DKD patients. Therefore, the effect of TSF on microalbuminuria could not be fully expressed under the intervention of ACEIs and ARBs. Moreover, it could be due to the large range in UAER (20–200 μg/min) in DKD patients with microalbuminuria stage, which could have generated larger standard deviations in both groups, resulting in negative statistical significance.

Although the mechanism of TSF in the treatment of DKD remains to be investigated in humans, studies have been done using the diabetic rat model. One study showed that TSF decreases UAER and reduces glomerulosclerotic index and interstitial ﬁbrotic index [19]. In another study, TSF exhibited a renal protective effect by improving lipid metabolism, correcting abnormal blood rheology parameters, inhibiting expression of TGF-β1 in renal tissue, enhancing expression of MMP-9, and reducing expression of collagen type IV [27]. Several individual herbs that comprise TSF have been investigated in clinical and laboratory studies for their effects against DKD. A meta-analysis by Li and colleagues [28] suggested that patients with DKD stages III–IV, who received astragalus injection (derived from *Astragalus membranaceus* (Fisch.) Bge.) at a dosage of 20–60 mL daily for 2–6 weeks, experienced improved renal function, decreased proteinuria and increased serum albumin compared with those in a control group. Researches on diabetic animal models have been conducted on individual herbs in TSF. Various studies on DKD animals have demonstrated that astragalus (*A*.*membranaceus* (Fisch.) Bge.) is capable of reducing albuminuria, improving renal function, and ameliorating pathological changes [29,30]. Burning bush (*E*.*alatus* (Thunb.) Sieb.) in the treatment of DKD rats for 12 weeks displayed a protective role in kidney injury [31]. A decoction made from rehmannia (*R*.*glutinosa*Libosch) was found in vitro to suppress advanced glycation end products induced by inflammation [32]. Catalpol, a chemical constituent extracted from rehmannia, improved renal function and reduced extracellular matrix accumulation in type 2 DKD rats [33]. Diosmin, a chemical constituent extracted from bitter orange (*C*.*aurantium* L.), can increase anti-oxidative stress markers in the kidneys of diabetic rats [34]. Iridoid, a total glycoside extracted fromcornus (*C*.*officinalis* Sieb. et Zuce), was found to efficiently decrease expression of renal fibrosis marker transforming growth factor beta 1 and matrices in DKD rats [35]. As the most active component of rhubarb (*R*.*palmatum* L.), rhein was found to reduce renal lesions and ameliorate dyslipidemia in diabetic rats [36]. As the main active component of notoginseng (*P*.*notoginseng* (Burk.) F.H. Chen), panax notoginseng saponins are patented for their anti-diabetic effects [37]. Notoginseng combined with astragalus can inhibit the proliferation of cultured glomerular mesangial cells [38].

ACEI/ARB agents have been widely used in DKD treatment. Their effectiveness in reducing or preventing microalbuminuria has been documented [8,9]. But a large number of DKD patients, who take ACEI/ARB medication have continued progression of 24h UP and inevitably develop ESRD. Studies of new medications have not yielded impressive results. Pyridorin, an advanced glycation end product inhibitor, did not reduce proteinuria after 1 year’s therapy [39]. Benfotiamine, a lipophilic thiamine derivative, did not reduce UAER after 12 weeks of treatment [40]. In the Sun-MACRO trial, sulodexide did not show efficacy in reducing macroalbuminuria [41]. Other new medications have serious side effects, such as avosentan [11] and aliskiren [42]. Even some established drugs such as thiazolidinediones, which reduce microalbuminuria [43] and macroalbuminuria [44], appear to increase the risk of heart failure [45] and bladder cancer [46,47]. Therefore, finding a new therapeutic strategy for patients with DKD is emergent.

The results of our study appear to indicate that TSF added to ACEI/ARB agents could reduce 24h UP to a level much lower than that of ACEI/ARB agents alone. Moreover, as eGFR is a marker of renal function, the renal protective properties of TSF induced improvement of eGFR levels in DKD patients. Compared with non-diabetic populations, adults with diabetes have the greatest decline in eGFR of 2.1–2.7 ml/min/1.73 m^2^/year [48], leading to end-stage renal disease within several years. eGFR decline can even occur despite treatment as in a 5-year study reported by Barnett et al., which found that eGFR decreased by −17.9 and −14.9 ml/min/1.73 m^2^ following therapy with telmisartan (an ARB) and enalapril (an ACEI) respectively [49]. Furthermore, in the 6-month AVOID trial, efficacy of aliskiren plus losartan and losartan alone in patients with stage1–3 CKD, patients in both groups displayed different degrees of eGFR decline [50]. Despite our trial having an intervention period of only 24 weeks, results revealed that eGFR increased in the TSF group while eGFR trended downward in the placebo group.

In our study, the proportions of adverse events did not show significant difference between TSF and placebo groups. There was only one severe adverse event in each group: One participant died of SAH in the TSF group and one participant died of AMI in the placebo group. These adverse events were not considered related to the study agent. Therefore, TSF appears to be a safe therapeutic treatment for DKD patients, but further evaluation is needed.

A limitation of our research is an intervention period of only 24 weeks. This study was not designed to observe hard end points of DKD, such as doubling of baseline serum creatinine concentration, ESRD, and death. The aim of this study was to evaluate efficacy of TSF in treating DKD patients with either microalbuminuria or macroalbuminuria. Sample size estimation was based on previous results of UAER change in patients with microalbuminuria, which consequently resulted in a diminished sample size in each stage. Moreover, to assess fully the renoprotective effect and safety of TSF, long-term follow-up is necessary.

In conclusion, TSF appears to decrease 24hurinary protein level and increase glomerular filtration rate in type 2 DKD patients with macroalbuminuria, although TSF did not significantly alter UAER in DKD patients with microalbuminuria. With favorable safety and efficacy, TSF may be an adjuvant therapy for treatment of DKD patients with macroalbuminuria.

## Supporting Information

S1 CONSORT Checklist CONSORT Checklist. (DOC)

S1 Fig High-performance liquid chromatography/mass spectrometry analysis of TSF. Representative total ion current (TIC) chromatograms of TSF obtained in negative ion electrospray. Visual inspection of the negative TIC plot indicates that the negative mode of ionization generated constituent information based on the ionizability of the compounds in TSF. The nine most representative compounds identified in TSF were: sweroside (peak 1), rhapontigenin (peak 2), isomucronulatol-7, 2'-di-glucoside (peak 3), naringin (peak 4), isonaringin (peak 5), melittoside (peak 6), ginsenoside Rg1 (peak 7), morroniside (peak 8), ginsenoside Rb1 (peak 9).
(TIF)

S1 Protocol Trial Protocol (English). (DOC)

S2 Protocol Trial Protocol (Chinese). (DOC)

S1 Table Chinese medicine symptomatology score survey instrument. (DOC)

S2 Table Nine most representative compounds in TSF identified by high-performance liquid chromatography/mass spectrometry. (DOC)

S3 Table WHOQOL-BREF scores in four domains and overall in microalbuminuria stage. (DOC)

S4 Table WHOQOL-BREF scores in four domains and overall in macroalbuminuria stage. (DOC)

S5 Table DQOL scores in four domains and overall in microalbuminuria stage. (DOC)

S6 Table DQOL scores in the four domains and overall in macroalbuminuria stage. (DOC)
