# Supplementary figures and images for: Efficacy and Safety of Tangshen Formula on Patients with Type 2 Diabetic Kidney Disease: A Multicenter Double-Blinded Randomized Placebo-Controlled Trial
Source: PLoS One. 2015 May 4;10(5):e0126027. doi: 10.1371/journal.pone.0126027 (PMC4418676; doi:10.1371/journal.pone.0126027)

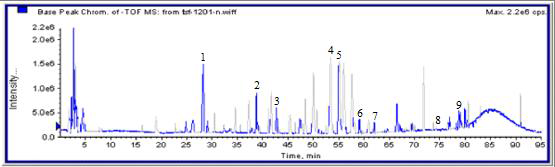

Supplement: S1 Fig — Representative total ion current (TIC) chromatograms of TSF obtained in negative ion electrospray. Visual inspection of the negative TIC plot indicates that the negative mode of ionization generated constituent information based on the ionizability of the compounds in TSF. The nine most representative compounds identified in TSF were: sweroside (peak 1), rhapontigenin (peak 2), isomucronulatol-7, 2'-di-glucoside (peak 3), naringin (peak 4), isonaringin (peak 5), melittoside (peak 6), ginsenoside Rg1 (peak 7), morroniside (peak 8), ginsenoside Rb1 (peak 9). (TIF) [file pone.0126027.s002.tif]
