# Supplementary material for: Efficacy and Safety of Tangshen Formula on Patients with Type 2 Diabetic Kidney Disease: A Multicenter Double-Blinded Randomized Placebo-Controlled Trial
Source: PLoS One. 2015 May 4;10(5):e0126027. doi: 10.1371/journal.pone.0126027 (PMC4418676; doi:10.1371/journal.pone.0126027)
Supplement: S1 Protocol — (DOC) [file pone.0126027.s003.doc]

**Clinical Trial Protocol of Chinese Herbal Granule Treats Type 2 Diabetic Kidney Disease**

——A Multicenter Double-Blinded Randomized Placebo-Controlled Trial

Investigational drug generic name: Tangshen Formula (TSF)

Unit in charge of the trial: China-Japan Friendship Hospital

Principal: Ping Li

Sponsor: China-Japan Friendship Hospital

Address: Institute of Clinical Medical Science, China-Japan Friendship Hospital, Yinghua DongLu, Hepingli Chaoyang District, Beijing 100029, P. R. China

Person in charge: Ping Li

Tel: +86-10-64227163

**Confidentiality statement**

This article contains confidential information belonging to the TSF research group of China-Japan Friendship Hospital. Disclosure to anyone other than the researchers, members of the Independent Ethics Committee / Institutional Review Board, without written permission of the TSF research group, is not allowed.

**Sponsor:** China-Japan Friendship Hospital

Address: Institute of Clinical Medical Science, China-Japan Friendship Hospital, Yinghua DongLu, Hepingli Chaoyang District, Beijing 100029, P. R. China

Person in charge: Ping Li Tel: +86-10-64227163

**Study uints**

Unit No 1. Department of Nephrology, Dongzhimen Hospital Affiliated to Beijing University of Chinese Medicine, Beijing.

Principal investigator: Jing Li Tel: 13671131943

Unit No 2. Department of Nephrology, Longhua Hospital, Shanghai University of TCM, Shanghai.

Principal investigator: Yueyi Deng Tel: 18917763063

Unit No 3. Department of Endocrinology, Hebei United University Affiliated Hospital, Tangshan.

Principal investigator: Xiuping Jin Tel: 13363365032

Unit No 4. Department of Endocrinology, Kailuan General Hospital, Tangshan

Principal investigator: Liping Shi Tel: 13503251077

Unit No 5. Department of Endocrinology, Tangshan Gongren Hospital, Tangshan

Principal investigator: Hui Fang Tel: 13831581838

**CRO:** Project Department of World Federation of Chines Medicine Societies

Clinical research associate: Yafeng Wang Tel: 86-10-58650378

**Data management:** Department of Pediatrics, the University of British Columbia

Statistics manager: Jean-Paul Collet E-mail: Jcollet@cw.bc.ca

**Contents**

**Table of Abbreviations 1**

**Background 2**

**Trial objectives 2**

**Study design 3**

Sample size 3

Randomization and concealment 3

Blindness 4

**Participants 6**

**Intervention 6**

**Outcome measurements 9**

**Adverse events 9**

**Quality control 10**

**References 11**

**Table of Abbreviations**

| **Abbreviations** | **English name** |
| --- | --- |
| ACEI | Angiotensin-Converting Enzyme Inhibitors |
| ADA | American Diabetes Association |
| AE | Adverse event |
| ALT | Alanine aminotransferase |
| ARB | Angiotensin Receptor Blocker |
| AST | Aspartate aminotransferase |
| BUN | Blood urea nitrogen |
| Cr | Creatinine |
| CRF | Case Report Form |
| DKD | Diabetic Jidney Disease |
| DM | Diabetic Mellitus |
| eGFR | estimated Glomerular Filtration Rate |
| FBG | Fasting Blood Glucose |
| HbA1C | Hemoglobin A1C |
| HDL-C | High Density Lipoprotein-Cholesterol |
| ICF | Informed Consent Forms |
| IEC | Independent Ethics Committee |
| LDL-C | Low Density Lipoprotein-Cholesterol |
| SAE | Serious Adverse Event |
| SFDA | China’s State Food and Drug Administration |
| SOP | Standard Operating Procedure |
| TC | Total Cholesterol |
| TCM | Traditional Chinese medicine |
| TG | Triglyceride |

**Background**

Diabetic mellitus (DM) is a lifelong progressive disease, usually associated with various complications in its late stage, which could cause great challenges and burdens to patients, their families, health care system and national economics. The prevalence of DM all over the world was 5.1% in 2003. The prevalence of DM in China also has a tendency to increase and reached to 5.5% in 2001[[[1]](#endnote-2)].

Diabetic kidney disease (DKD) is one of the most common microvascular complications of diabetes mellitus, and it has become the second cause of end-stage renal disease (ESRD) in China [[[2]](#endnote-3)]. Up to now, there is no proved therapy to cure or prevent the progression of DKD, though some reports showed that good management of blood glucose, blood pressure and blood lipid and medicine application of Angiotensin-converting enzyme inhibitors（ACEI）or Angiotensin Receptor blockers (ARB) might postpone the disease’ progression [[[3]](#endnote-4),[[4]](#endnote-5)]. Traditional Chinese medicine (TCM) has been applied to treat DKD for a long history, and recently several studies have reported some advantages of TCM in ameliorating clinical symptoms, improving quality of life, decreasing urinary protein excretion and improving renal function [[[5]](#endnote-6)-,[[6]](#endnote-7),[[7]](#endnote-8)]. Nevertheless, these trials of either small sample size or lack of placebo-controlled arm or low quality monitoring and management of study process take high risk bias to these evidences to support the effects of TCM. Hence, it is necessary and urgent to conduct a well-designed high quality RCT to assess the efficacy and safety of TCM for the treatment of DKD patients.

**Trial objectives**

To evaluate the efficacy and safety of TSF in treating diabetic kidney disease.

**Study design**

The study is designed as a randomized, double-blinded, placebo-controlled trial. It will be conducted in the following six hospitals: China-Japan Friendship Hospital, Bejing; Longhua Hospital affiliated to Shanghai University of TCM, Shanghai; Dongzhimen Hospital affiliated to Beijing University of Chinese Medicine; Hebei United University School of Medicine Affiliated Hospital, Tangshan; Kailuan Hospital, Tangshan; and Tangshan Workers Hospital, Tangshan. Figure 1 is a flow chat of this trial.

**Sample size**

The sample size has been calculated according to our preliminary results [[[8]](#endnote-9)]. The mean reduction of UAER in the control group was 30.19, and that in TSF group was 81.67. The ratio of patients between the treatment group and the placebo group is designed as 2:1. The sample size has been estimated to be 56 in the control group, and 112 in the treatment group. Assuming a dropout rate of 15%, 90% power to detect and a 0.05 two-side level of significance, the sample size of DKD patients has been estimated to be 192.

**Randomization and concealment**

The randomization of the trial will be completed by an independent clinical research coordinator, project department of World Federation of Chinese Medicine Societies. 192 seeds and 6 blocks were set to generate the random allocation sequence according to the method of blocked randomization. Investigators of each center sequentially enroll patients according to their consultant order. The randomization sequence will be kept by project department of World Federation of Chinese Medicine Societies, and it will be disclosed after the trial finally finished except for emergency during this trial (such as severe adverse event). Drug administrator in every center is an independent researcher, who is in charge of supervision, dissemination and compliance recording of the drugs. They have the random sequence of their own center in order to dispense drugs

**Blindness**

Double-blinding method is adopted in this study. As the package, color, smell and taste of the placebo is the same as TSF, so researchers and participants won’t know the kind of medicine and group allocation. It was not allowed for the drug administrator to neither contact with investigators, nor exchange information with patients. They are trained how to illustrate the usage and dose of this formula for the patients but not to convey any treatment information to participants.

The outcome assessment will be conducted by independent researchers who don’t know the group allocation of patients.

Figure 1: The flowchart of the trial:

Screening for type 2 diabetic kidney disease with microalbuminuia /marcoalbuminuia individuals

Sign consent

Inclusion criteria achieved?

Run-in period (2 weeks)

NO

Excluded

Primary outcome:

UAER & 24h urinary protein

Secondary outcome (1):

Scr，BUN， eGFR

Blood lipid

Secondary outcome (2):

TCM syndrome & quality of life

Completed the 24-wk administration

**TSF group**：

TSF+ACEI/ARB

**Placebo group**： Placebo + ACEI/ARB

Visits and clinical measurements, medication

at 4,8,12,16,20,24 weeks

YES

Randomization

**Patient ratio**

**2 : 1**

**Participants**

**Diagnosis criteria**

Patients with type 2 diabetes are diagnosed according to the American Diabetes Association criteria [[[9]](#endnote-10)],and micro-albuminuria and macro-albuminuria stages of DKD are defined in the light of the K-DOQI guidelines [[[10]](#endnote-11)], and the Chinese syndrome differentiation type as Qi and Yin deficiency with blood stasis is based on the guiding principle of clinical research on new drugs of traditional Chinese medicine [[[11]](#endnote-12)].

**Inclusion and exclusion criteria**

Patients aged 25-75 years who have been diagnosed as type 2 diabetes and DKD will be included in the study and meet the following inclusion criteria: the Chinese syndrome differentiation type as Qi and Yin deficiency with blood stasis; blood pressure < 140/90mmHg; fasting blood glucose (FBG) ≤ 7.8 mmol/L and glycosylated hemoglobin (HbA1C) ≤7.5%; UAER>20 μg/min , and/or 24-hour urinary protein level 0.5~2.0 g, and estimated glomerular filtration rate (eGFR) estimated by Cockcroft-Grault equation ranging from 60ml/min to130ml/min; signed written informed consent.

Patients will be excluded if they had a history of primary kidney diseases or systematic disease with elevated urinary protein. Other major exclusion criteria were other endocrine or metabolic disease; mental disorders; women in pregnancy or lactation; herb allergy; a history of myocardial infarction, angina pectoris or other recent cardiovascular problems (including cerebrovascular events) within the last 3 months or recent infection within the last 4 weeks; fasting serum triglyceride > 10 mmol/L; impaired hepatic function, defined as Alanine transaminase (ALT) and/or Aspartate aminotransferase (AST) level≥2 times the upper limit of the nomal reference range. Patients who take glucocorticosteroid, thiazide diuretics and niacin within the last 3 months will also be excluded.

**Intervention**

**Ingredients of TSF**： TSF consists of radix astragali (Huangqi) 30 grams, rehmannia dride rhizome (Shengdi) 12 grams, prepared rhubarb (Zhidahuang) 6 grams, notoginseng (Sanqi) 3 grams, evonymus alata (Weimao)15 grams, medicinal cornel (Shanzhuyu) 9 grams and fructus aurantii (Zhiqiao) 10 grams in dried raw material. TSF is manufactured by Jiangyin Tianjiang Pharmaceutical Company Limited (Lot No. 0606320), and restored at 15℃ -30℃.

**Ingredients of placebo:** lactose, dextrin and artificial food coloring. Placebo is also manufactured by Jiangyin Tianjiang Pharmaceutical Company Limited.

**Allocation of drugs:** Drugs with specific codes will be assigned to each hospital, due to the allocation design for each unit. Each study unit designates an experimental drug administrator. The researchers will screen qualified subjects. After subjects sign informed consent and complete the run-in period, the drug administrator will allocate drugs according to the code order and record the allocating information in the "clinical trial drug use record" form. The researchers will in charge of recording how many packages of drugs are allocated by the drug administrator, taken by the participant and returned to the uint, at each visit of a participant. The unused drugs will be returned to sponsor and destroyed according to certain procedure.

**Basic treatments:** All participants will be instructed to keep a low-salt diabetic diet, including a reasonable match of calories, protein, carbohydrates and fat, and keep a fixed level of exercise based on their age, urine protein level and cardiac function. According to ADA’s guideline, all participants will receive either an ACEI or ARB agent treatment. Antihypertensive treatment, glycemic control and antilipemic agents were adopted as conventional treatments. All the western drugs will be given open-labelled.

**Treatment for TSF group:** In addition to the basic treatments, each participant in the intervention group will be prescribed TSF. TSF will be administered to participants with two packages (8 grams) per time and twice a day.

**Treatment for placebo group:** In addition to the basic treatments, each participant in the placebo group will receive placebo medicine with the same appearance, shape and smell as TSF. Furthermore, the method and time of taking drugs will be the same as that in the treatment group.

**Duration of intervention:** All participants have to visit researchers every 4 weeks with a total of 24 weeks. Table 1 is an overview of specific measurements in each visit time.

Table 1: The content of data capture

| Item | Screening period (unit:weeks) | Intervention period (unit:weeks) | | | | | | |
| --- | --- | --- | --- | --- | --- | --- | --- | --- |
|  | -2 | 0 | 4 | 8 | 12 | 16 | 20 | 24 |
| Record of medical history | √ |  |  |  |  |  |  |  |
| General physical examination | √ | √ | √ | √ | √ | √ | √ | √ |
| Blood and urine regulation | √ | √ |  |  | √ |  |  | √ |
| Electrocardiogram | √ | √ |  |  | √ |  |  | √ |
| ALT, AST | √ | √ |  |  | √ |  |  | √ |
| BUN, Cr, eGFR | √ | √ |  |  | √ |  |  | √ |
| HbA1c | √ | √ |  |  | √ |  |  | √ |
| UAER &24h urinary protein | √ | √ |  |  | √ |  |  | √ |
| serum TC, TG, LDL, VLDL | √ | √ |  |  | √ |  |  | √ |
| WHO QOL-BREF, DQOL |  | √ |  |  | √ |  |  | √ |
| Evaluation of TCM Syndrome | √ | √ | √ | √ | √ | √ | √ | √ |
| Drug Distribution and record |  | √ | √ | √ | √ | √ | √ |  |
| Left drugs Retrieval |  |  | √ | √ | √ | √ | √ | √ |
| Adverse events |  | √ | √ | √ | √ | √ | √ | √ |

√：Item need to be done during different period

**Outcome measurements**

**Primary outcomes**

UAER for DKD patients in the microalbuminuria stage and 24-hour urinary protein excretion amount for DKD patients in the macroalbuminuria stage are considered as the primary outcome measurements.

**Secondary outcomes**

The following domains are recognized as the secondary outcome measurements, including: renal function, regarding serum creatinine (Scr), blood urinary nitrogen（BUN）and eGFR measured by Cockcroft-Grault equation with a 0.85 correction factor for women; blood lipid, containing total cholesterol (TC), triglyceride (TG), high density lipoprotein-cholesterol (HDL-C)and low density lipoprotein-cholesterol (LDL-C); glycemic management (assessment by HbA1c); the improvement of TCM symptoms (evaluation by TCM symptomatology scores; the improvement of quality of life (QOL) (assessment by WHO QOL-BREF and Diabetic Scall of Quality of Life of Chinese).

**Safety assessment**

The results of blood, urine routine tests, electrocardiogram (ECG), ALT and AST tests will be the comprehensive reflection of safety assessment.

**Adverse events**

Adverse events (AE) refer to adverse medical events occur after treatment with a drug, which do not necessarily have a causal relationship with the treatment. Serious adverse events (SAE) include death, hospitalization or prolonged length of hospitalization, disability and congenital malformations. We have established the adverse event monitoring system and defined the three-grade criteria of adverse events: the first level as it could be endured or only mild discomfort with no affection of daily life; the second level as it causes discomfort and affects daily life; the third level as it causes handicap to self-care ability.

**Possible adverse events due to TSF:** TSF contains the herbal medicine rhubarb, which may lead to the following adverse events to participants: 1) Diarrhea and secondary constipation: Loose stools and diarrhea are the most common adverse reactions of rhubarb. Severe cases may have symptoms of dehydration, such as sunken eyes, dry lips and fatigue. Tannic acid in rhubarb has anti-diarrhea action. Taking rhubarb for a long time can cause stress reaction reduction of intestinal nerve cell, leading to rhubarb dependent constipation. 2) Melanosis coli: Herbal medicine with strong cathartic actions usually contains anthraquinone derivatives, which may cause colon melanosis coli. Diagnosis of melanosis coli is mainly based on microscopic examination and biopsy specimens. Taking rhubarb for a long-term or with a high dosage can increase the secretion of intestinal mucus glands, make intestinal epithelial cells produce more histocompatibility complex, and increase macrophage activity and chemotaxis. Then the macrophage phagocytosis is strengthened, causing colon melanosis coli. 3) Other adverse events: A few cases reported that application of rhubarb might cause elevated ALT and serum creatinine, which were mainly due to excessive intake without instruction of a physician.

**Possible adverse events due to ACEIs/ARBs:** The main side effects of ACEIs and ARBs are dry cough, rise of serum potassium and rise of serum creatinine.

**Quality control**

TCM doctors participating in this trial are required to be attending physician or higher in title with relatively abundant clinical experience and have enough time and energy to be dedicated to this clinical research. All the investigators including TCM doctors, drug administrators and outcome assessors have trained of the Standing Operating Procedure (SOP) in order to get a thorough understanding of the protocol and the principal parameters of the study. An auditor has arranged to monitor the process of study, supervise the study progression and check the reliability of records. Moreover, we have set up an adverse event monitoring system. The type, starting and ending time, clinical manifestation, treatment process, and prognosis of adverse effects will be well-recorded. The relationship of adverse effects with drugs will be assessed according to the 5 grades formulated by the monitor center of the Ministry of health of China, including certainly related, probably related, probably unrelated, certainly unrelated and unable to judge. The certainly related or probably related adverse effects will be recognized as drug related adverse effects.

**Reference**

1. . Gu D, Reynolds K, Duan X, Xin X, Chen J, Wu X, et al. Prevalence of diabetes and impaired fasting glucose in the Chinese adult population: International Collaborative Study of Cardiovascular Disease in Asia (InterASIA). Diabetologia 2003; 46:1190-8 [↑](#endnote-ref-2)
2. . Dialysis and Transplantation Registration Group, Chinese Society of Nephrology. The report about the registration of dialysis and transplantation in China 1999. Chin J Nephrol 2001; 17: 77-8. [↑](#endnote-ref-3)
3. . Ruggenenti P, Fassi A, Ilieva A P, Bruno S, Iliev I P, Bruseqan V, et al. Preventing microalbuminuria in type 2 diabetes. N Engl J Med. 2004;351:1941-51. [↑](#endnote-ref-4)
4. . Barry MB, Mark EC, Dick DE Z, William FK, William EM, Hans-Henrik P, et al. Effects of Losartan on renal and cardiovascular out comes in patients with type 2 diabetes and nephropathy. N Engl J Med. 2001; 345: 861-869. [↑](#endnote-ref-5)
5. . Zhang LF, Zhao JX, Lv RH, Yang HT, Cao SL, Huang XM, et al. Study of Effectiveness and Safety of Optimum Program for Prevention and Treatment of Renal Insufficiency of Diabetic Nephropathy. Journal of Traditional Chinese Medicine(chin). 2006; 47: 755-758. [↑](#endnote-ref-6)
6. . Zhao J, Mu X, Wang S, Song M, Huang X, Yu X, et al. Clinical Observation on 31 Case of Diabetic Nephropathy with Renal Insufficiency at Compensatory Phase Treated with Zhixiao Wwenshenning Granule. Journal of Traditional Chinese Medicine. 2005; 46:677-679. Chinese. [↑](#endnote-ref-7)
7. . Song M, Yang M, Mu X, Zhang L, Zhao J. Evaluation of curative effects of Chinese medical therapies on renal function and symptoms of renal failure of diabetic nephropathy. Journal of Beijing University of TraditionaI Chinese Medicine. 2006;29: 429-432. Chinese. [↑](#endnote-ref-8)
8. . Feng JC, Ni Q. Clinical observation on treatment of diabetic nephropathy by Tangshen capsule. Chinese Journal of Integrative Medicine. 2000;20: 212-214. Chinese. [↑](#endnote-ref-9)
9. . American Diabetes Association. Standards of Medical Care in Diabetes: 2006. Diabetes Care. 2006;29: 4-42. [↑](#endnote-ref-10)
10. . National Kidney Foundation. KDOQI Clinical Practice Guidelines and Clinical Practice Recommendations for Diabetes and Chronic Kidney Disease. Am J Kidney Dis. 2007; 49:14-46. [↑](#endnote-ref-11)
11. . Zheng XY (2002) clinical research guidelinesfornew investigational drugs in traditional Chinese medicine. Beijing: China Medical Science Press163-168. Chinese. [↑](#endnote-ref-12)
