# Supplementary material for: Efficacy and Safety of Tangshen Formula on Patients with Type 2 Diabetic Kidney Disease: A Multicenter Double-Blinded Randomized Placebo-Controlled Trial
Source: PLoS One. 2015 May 4;10(5):e0126027. doi: 10.1371/journal.pone.0126027 (PMC4418676; doi:10.1371/journal.pone.0126027)
Supplement: S2 Protocol — (DOC) [file pone.0126027.s004.doc]

**国家973项目中医理论专项：项目编号2005CB523503**

**首都医学发展基金联合攻关项目：项目编号2005-SF-1-D15**

糖肾方治疗糖尿病肾病有效性和安全性的随机双盲、

安慰剂平行对照、多中心临床试验方案

试验药物通用名：糖肾方

临床研究负责单位：中日友好医院

负责人：李平

申办单位：中日友好医院

地址：北京市朝阳区和平里樱花东街中日友好医院临床医学研究所

邮编：100029

课题负责人：李平

联系电话：010-64227163

**保密声明**

本文包含属于中日友好医院糖肾方课题组所有的保密资料。未经中日友好医院糖肾方课题组的书面许可，不得将本文泄露给除研究人员、独立伦理委员会/机构审查委员会成员以外的其他人，除非在法律或规章制度的许可范围内。

临床研究单位、统计分析单位

**申办及临床研究负责单位：**

**中日友好医院**

地址：北京市朝阳区和平里樱花东街2号 邮编：100029

项目负责人：李平，联系电话：010-64227163

**临床研究参加单位：**

**北京中医药大学东直门医院肾病科**

主要研究者：李靖，联系电话：13671131943

**上海中医药大学附属龙华医院肾内科**

主要研究者：邓跃毅，联系电话：18917763063

**[[1]](#footnote-2)华北煤炭医学院附属医院内分泌科**

主要研究者：金秀平，联系电话：13363365032

**开滦医院内分泌科**

主要研究者：史丽萍，联系电话：13503251077

**唐山工人医院内分泌科**

主要研究者：房辉，联系电话：13831581838

**监查单位：**

**世界中医药学会联合会项目部**

项目负责人：王亚锋 联系电话：010-58650378

**数据管理与统计分析：**

**加拿大UBC大学**

主要负责人：Jean-Paul Collet E-mail: Jcollet@cw.bc.ca

**目录**

[方案摘要 1](#__RefHeading___Toc414865311)

[缩略语表 3](#__RefHeading___Toc414865312)

[1 研究背景 4](#__RefHeading___Toc414865313)

[2 研究目的 4](#__RefHeading___Toc414865314)

[3 研究总体设计 5](#__RefHeading___Toc414865315)

[3.1 试验设计方法 5](#__RefHeading___Toc414865316)

[3.2 样本量 5](#__RefHeading___Toc414865317)

[3.3 随机分组和随机化隐藏 5](#__RefHeading___Toc414865318)

[3.4 盲法 5](#__RefHeading___Toc414865319)

[4 受试者选择 6](#__RefHeading___Toc414865320)

[4.1西医诊断标准 6](#__RefHeading___Toc414865321)

[4.2 中医辨证 6](#__RefHeading___Toc414865322)

[4.3 中医证候计分标准 6](#__RefHeading___Toc414865323)

[4.4纳入病例标准 7](#__RefHeading___Toc414865324)

[4.5 排除标准 8](#__RefHeading___Toc414865325)

[5 治疗方案 8](#__RefHeading___Toc414865326)

[5.1 试验药物 8](#__RefHeading___Toc414865327)

[5.2 药物的包装 8](#__RefHeading___Toc414865328)

[5.3 药物分配及记录 9](#__RefHeading___Toc414865329)

[5.4 给药方法 9](#__RefHeading___Toc414865330)

[5.5 试验期间合并用药的规定 9](#__RefHeading___Toc414865331)

[5.6 药品清点 10](#__RefHeading___Toc414865332)

[5.7 药品保存与发放 10](#__RefHeading___Toc414865333)

[5.8 受试者退出试验的条件及步骤 10](#__RefHeading___Toc414865334)

[5.9 剔除及脱落病例标准 10](#__RefHeading___Toc414865335)

[6 有效性及安全性评价 11](#__RefHeading___Toc414865336)

[7 不良事件监控 11](#__RefHeading___Toc414865337)

[7.1 定义 11](#__RefHeading___Toc414865338)

[7.2严重程度的判断 12](#__RefHeading___Toc414865339)

[7.3本研究预期可能发生的不良反应： 12](#__RefHeading___Toc414865340)

[8.4 与药物因果关系判断 13](#__RefHeading___Toc414865341)

[8.4.1 不良反应判断的因果判断指标 13](#__RefHeading___Toc414865342)

[8.4.2 因果判定标准 13](#__RefHeading___Toc414865343)

[8.5不良事件处理 13](#__RefHeading___Toc414865344)

[8.5.1报告方法 13](#__RefHeading___Toc414865345)

[8.5.2 处理程序 13](#__RefHeading___Toc414865346)

[8.5.3 严重不良事件的处理 13](#__RefHeading___Toc414865347)

[8.5.4 随访未缓解的不良事件 13](#__RefHeading___Toc414865348)

[9 数据管理 14](#__RefHeading___Toc414865349)

[9.1 填写CRF 14](#__RefHeading___Toc414865350)

[9.2 核对CRF 14](#__RefHeading___Toc414865351)

[9.3 收回CRF 14](#__RefHeading___Toc414865352)

[9.4 发疑问表 14](#__RefHeading___Toc414865353)

[9.5 建立数据库 14](#__RefHeading___Toc414865354)

[9.6 数据录入 14](#__RefHeading___Toc414865355)

[9.7 数据库核查 14](#__RefHeading___Toc414865356)

[9.8 数据备份 15](#__RefHeading___Toc414865357)

[9.9 盲态审核与揭盲规定 15](#__RefHeading___Toc414865358)

[10 统计分析 15](#__RefHeading___Toc414865359)

[10.1 一般原则 15](#__RefHeading___Toc414865360)

[10.2 统计分析人群 15](#__RefHeading___Toc414865361)

[10.3 统计分析方法 16](#__RefHeading___Toc414865362)

[10.3.1 入选及完成情况 16](#__RefHeading___Toc414865363)

[10.3.2 基线均衡性分析 16](#__RefHeading___Toc414865364)

[10.3.3 影响试验评价因素比较 16](#__RefHeading___Toc414865365)

[10.3.4 有效性分析 16](#__RefHeading___Toc414865366)

[10.3.5 安全性分析 16](#__RefHeading___Toc414865367)

[11 质量控制 17](#__RefHeading___Toc414865368)

[12 伦理原则 17](#__RefHeading___Toc414865369)

[12.1 伦理委员会审批试验方案 17](#__RefHeading___Toc414865370)

[12.2 受益与风险 17](#__RefHeading___Toc414865371)

[12.3 受试者的医疗和保护 18](#__RefHeading___Toc414865372)

[12.4受试者隐私的保护 18](#__RefHeading___Toc414865373)

[12.5 知情同意的过程 18](#__RefHeading___Toc414865374)

[12.6 伦理学要求 18](#__RefHeading___Toc414865375)

[13 临床研究预期进度和完成日期 19](#__RefHeading___Toc414865376)

[13.1 临床研究开始时间 19](#__RefHeading___Toc414865377)

[13.2 临床研究完成时间 19](#__RefHeading___Toc414865378)

[附表 20](#__RefHeading___Toc414865379)

[附表一 临床试验研究流程图 20](#__RefHeading___Toc414865380)

[附表二 试验用药品使用记录表 21](#__RefHeading___Toc414865381)

[附表三 严重不良事件报告表(SAE) 22](#__RefHeading___Toc414865382)

# 方案摘要

**研究题目**：糖肾方治疗糖尿病肾病有效性和安全性的随机、双盲、安慰剂平行对照、多中心临床试验。

**研究目的**：评价糖肾方治疗糖尿病肾病（2型糖尿病）的有效性和安全性。

**总体设计**：随机、双盲、安慰剂平行对照、多中心临床试验。

本研究采用加载试验设计，两组受试者在西医规范降尿蛋白、降血糖、降血压、降血脂治疗的基础上，采用安慰剂对照。

**研究单位**：中日友好医院

北京中医药大学东直门医院

上海中医药大学附属龙华医院

华北煤炭医学院附属医院

开滦医院

唐山工人医院

**受试人群**：符合2型糖尿病肾病诊断（Mogensen分期）。Ⅲ期糖尿病肾病尿白蛋白尿排泄率（urinary albumin excrection, UAER）20～200μg/min ；IV期糖尿病肾病24小时蛋白尿0.5~2.0 g/日；eGFR 130～60 ml/min。中医辨证为气阴两虚夹瘀证。年龄在25～75岁，性别不限。

**样本量**：192例（试验组128例、对照组64例）。

**治疗方案**：

**⑴基础治疗方案**

1. 降尿蛋白，保护肾功能：ACEI/ARB类药物，个体化选择其中一种，口服。
2. 降血压、降糖、降血脂：按照2006年ADA发布的指南合理选择用药。

**⑵试验组**

在上述基础治疗方案基础上，给予糖肾方颗粒。

糖肾方配方颗粒：每次8g，每日2次，早、晚服用，开水冲服。

**⑶对照组**

在上述基础治疗方案基础上，给予安慰剂。

安慰剂：每次8g，每日2次，早、晚服用，开水冲服。

**⑷给药周期**

给药观察期24周。

**有效性评价指标：**

**主要疗效指标**

糖尿病肾病Ⅲ期以UAER为主要疗效指标，Ⅳ的主要疗效指标为24小时尿蛋白定量。筛选期、基线、给药12、24周各检查1次。

**次要疗效指标**

1. 肾功能：血清肌酐、BUN、肾小球滤过率（采用Cockcroft-Grault公式估算）；
2. 血脂：总胆固醇、甘油三酯、高密度脂蛋白胆固醇、低密度脂蛋白胆固醇；
3. 中医症状评分：按症状无、轻、中、重评分；
4. 生存质量评价：采用WHO QOL-BREF和糖尿病特异生存质量量表进行。

注：各指标在筛选期、基线、给药12、24周各检查1次。

**安全性指标**

1. 血、尿常规，心电图，ALT、AST。筛选期、基线、给药12、24周各检查1次。
2. 不良事件：随时详细记录。

**研究周期：**

导入期2周，给药观察期 24周。

# 缩略语表

| **缩写** | **英文全称** | **中文全称** |
| --- | --- | --- |
| ACEI | Angiotensin-Converting Enzyme Inhibitors | 血管紧张素转化酶抑制剂 |
| ADA | American Diabetes Association | 美国糖尿病协会 |
| AE | Adverse event | 不良反应 |
| ALT | Alanine aminotransferase | 丙氨酸氨基转移酶 |
| ARB | Angiotensin Receptor Blocker | 血管紧张素受体拮抗剂 |
| AST | Aspartate aminotransferase | 天门冬氨酸氨基转移酶 |
| BUN | Blood urea nitrogen | 血尿素氮 |
| Cr | Creatinine | 肌酐 |
| CRF | Case Report Form | 病例报告表 |
| DM | diabetic mellitus | 糖尿病 |
| DN | diabetic nephropathy | 糖尿病肾病 |
| eGFR | estimated Glomerular Filtration Rate | 估算肾小球滤过率 |
| FBG | Fasting Blood Glucose | 空腹血糖 |
| HbA1C | Hemoglobin A1C | 糖化血红蛋白 |
| HDL | High Density Lipoprotein | 高密度脂蛋白 |
| ICF | Informed consent forms | 知情同意书 |
| IEC | Independent Ethics Committee | 独立伦理委员会 |
| LDL | Low Density Lipoprotein | 低密度脂蛋白 |
| SAE | Serious Adverse Event | 严重不良事件 |
| SFDA | China’s State Food and Drug Administration | 中国国家食品药品监督管理局 |
| SOP | Standard Operating Procedure | 标准操作程序 |
| TC | Total Cholesterol | 总胆固醇 |
| TCM | Traditional Chinese medicine | 中医 |
| TG | Triglyceride | 甘油三酯 |

糖肾方治疗糖尿病肾病有效性和安全性的

随机双盲、安慰剂平行对照、多中心临床试验方案

# 1 研究背景

糖尿病，作为一种终生进展性疾病，在其晚期阶段，因常常伴发多种并发症，而给患者及其家庭、医疗机构和国家带来了巨大的挑战和经济负担。全球糖尿病患病率2003年为5.1%， 2001年的流行病学调查显示中国糖尿病的患病率已达5.5% [[[2]](#endnote-2)]。

糖尿病肾病（diabetic nephropathy, DN）, 作为糖尿病最常见的微血管并发症，在中国已成为导致终末期肾脏疾病的第二位病因[[[3]](#endnote-3)]。虽然有部分研究表明，良好的血糖、血压、血脂管理和使用血管紧张素转化酶抑制剂（Angiotensin-converting enzyme inhibitors，ACEI）或血管紧张素受体拮抗剂（Angiotensin Receptor blockers，ARB）可以延缓其进展[[[4]](#endnote-4),[[5]](#endnote-5)]。但到目前为止，仍未有公认的可以防治DN的有效方法。中医药治疗DN由来已久。当前一些研究也表明中医药疗法能改善DN患者临床症状、减少尿蛋白排泄、改善肾功能、提高生存质量[[[6]](#endnote-6)-,[[7]](#endnote-7),[[8]](#endnote-8)]。但是，这些研究往往存在一些质量缺陷，例如：研究样本量过小、缺乏安慰剂对照、临床研究质量管理缺如等等，导致了其研究结果可信度不足。所以，急需开展高质量的中药复方干预DN的临床研究，以为中药治疗DN的有效性和安全性提供证据。

糖肾方是由现代名老中医时振声教授治疗糖尿病肾病临床经验方总结归纳而成，由黄芪、生地、大黄等七味中药组成，具有益气滋阴，活血通络的功效。前期小样本临床观察发现，在糖尿病肾病的治疗过程中可显著降低尿微量白蛋白、保护肾功能和改善中医症状[[[9]](#endnote-9)]。本试验拟在国家973项目中医理论专项（2005CB523503）、首都发展基金联合攻关项目（2005-SF-1-D15）的支持下，科学评价糖肾方治疗糖尿病肾病的临床疗效和安全性。

# 2 研究目的

评价糖肾方治疗糖尿病肾病的有效性和安全性，为治疗糖尿病肾病的新药研发和机制探讨提供临床依据。

# 3 研究总体设计

## 3.1 试验设计方法

采用随机、双盲、安慰剂平行对照、多中心临床试验设计。各中心包括：中日友好医院、北京中医药大学东直门医院、上海中医药大学附属龙华医院、唐山工人医院、华北煤炭医学院附属医院、开滦医院。

## 3.2 样本量

样本量估算（试验组Vs安慰剂组）**--根据尿蛋白排泄率**

根据既往糖肾方的临床试验结果^[Error: Reference source not found]^：实验结束后，尿蛋白排泄率在对照组下降30.19μg/min，在糖肾方组下降81.67μg/min。糖肾方组和安慰剂组的病例数比例设置为2:1。在α=0.05,β=0.1的水平下，研究的样本含量根据统计学公式测算为安慰剂组56例，糖肾方组112例，考虑不超过15%的退出率，总例数确定为192例，安慰剂组64例，糖肾方组128例。

## 3.3 随机分组和随机化隐藏

随机序列的产生及随机分组由独立的临床研究机构——世界中医药学会联合会的项目部完成。采用区组随机方法，借助SPSS 10统计软件，给定种子数，产生192例（试验组、对照组）受试者所接受处理的随机安排，列出流水号为001～192所对应的治疗分配（随机编码表）。北京中医药大学东直门医院36例、上海中医药大学龙华医院42例、唐山工人医院36例、中日友好医院42例、华北煤炭医学院附属医院24例、开滦医院12例。随机方案由世界中医药学会联合会的项目部统一保存盲底，若在试验开展过程中发生紧急事件（如严重不良事件），经本研究负责人同意后，可以揭盲。试验结束后由世界中医药学会联合会项目部统一揭盲。

## 3.4 盲法

本试验采用双盲设计，制做安慰剂，其外观与糖肾方一致。试验药物和安慰剂均由江苏省江阴天江药业有限公司提供外包装。试验药物和对照药物采用相同的外包装盒和标签。各中心实验药物管理员将接受统一规范的培训，以使他向患者解释清楚具体服药方法和剂量。临床疗效的评估由独立的研究成员在不知晓患者分组信息的情况下完成。

临床观察结束后，数据的统计分析由独立的统计学专家在不知晓各组的用药信息（糖肾方组、安慰剂组）情况下完成。

# 4 受试者选择

## 4.1西医诊断标准

**糖尿病**

采用2006年美国糖尿病协会发布的“糖尿病诊疗标准”[[[10]](#endnote-10)]：

①糖尿病症状+任意时间血浆葡萄糖（葡萄糖氧化酶法）水平>11.1mmol/L（200mg/dl）；或②空腹血浆葡萄糖（FBG）水平>7.0mmol/L（126mg/dl）；或③OGTT试验中，2hBG水平>11.1mmol/L（200mg/dl）。

**糖尿病肾病**

参考Mogensen分期标准[[[11]](#endnote-11)]： III期糖尿病肾病尿白蛋白尿排泄率（UAER）在20～200μg/min；IV期糖尿病肾病24小时尿蛋白定量＞0.5g/日，GFR正常或减低。

## 4.2 中医辨证

参考2002年颁布的《中药新药临床研究指导原则》[[[12]](#endnote-12)]

**气阴两虚夹瘀证：**

主症：倦怠乏力，腰膝酸软，手足心热，咽干口燥，气短懒言

次症：易患感冒，面色无华，心烦，肢体麻木，浮肿，夜尿频，便秘，溲赤。

舌象：舌质暗红，或舌有瘀斑瘀点，或见舌下静脉曲张；少苔、无苔、苔薄黄或薄白

脉象：脉细、弦细或细数

注：满足主证至少两项、次证两项以上方可诊断。

## 4.3 中医证候积分标准

| 倦怠乏力 | □0分：正常。  □2分：不耐劳力 。  □4分：可坚持轻体力劳动。  □6分：勉强支持日常活动。 |
| --- | --- |
| 腰膝酸软 | □0分：正常。  □2分：腰膝酸软，时而作痛。  □4分：隐隐酸软，须常变换体位。  □6分：腰痛如折，持续不已，须服药可缓解。 |
| 手足心热 | □0分：正常。  □2分：手足心发热，时有时无。  □4分：手足心发热，手足需暴露。  □6分：手足心发烫，欲接触冷物。 |
| 咽干口燥 | □0分：正常。  □2分：口咽微干。  □4分：口咽干燥少津。  □6分：口咽干燥欲饮。 |
| 气短懒言 | □0分：正常。  □2分：劳累后气短。  □4分：一般活动即气短。  □6分：懒言，不活动也气短。 |
| 易患感冒 | □0分：正常。  □1分：每年感冒6次以上。  □2分：每年感冒10次以上。  □3分：每年感冒12次以上。 |
| 面色无华 | □0分：正常。  □1分：淡白。  □2分：淡白无华。  □3分：苍白或萎黄。 |
| 心烦 | □0分：正常。  □1分：偶有心烦。  □2分：烦躁不宁，尚能自控。  □3分：烦躁不宁，难以自控。 |
| 肢体麻木 | □0分：正常。  □1分：手足麻木。  □2分：四肢麻木。  □3分：全身麻木。 |
| 浮肿 | □0分：正常。  □1分：晨起眼睑浮肿，或午后足肿，肿势隐约可见 。  □2分：眼睑及双下肢浮肿，按之有陷 。  □3分：全身浮肿，按之深陷。 |
| 夜尿频 | □0分：正常。  □1分：夜尿2次。  □2分：夜尿3-4次。  □3分：夜尿5次以上。 |
| 便秘 | □0分：正常。  □1分：排便硬而费力。  □2分：大便硬结，2-3天一行。  □3分：大便硬结，3天以上一行。 |
| 溲赤 | □0分：无  □1分：有。 |
| 舌象 | 舌质：□舌质暗红 □舌有瘀斑瘀点 □舌下静脉曲张 □其它 □正常  □苔：□少苔 □无苔 □薄黄 □薄白 □其它 □正常 |
| 脉象 | □细 □弦细 □细数 □其它 □正常 |

## 4.4纳入病例标准

⑴符合糖尿病肾病诊断；

⑵中医辨证为气阴两虚夹瘀证；

⑶UAER＞20μg/min , 和/或24h尿蛋白定量0.5～2.0g；

⑷估算肾小球滤过率（Cockcroft-Grault公式）60ml/min～130ml/min；

⑸血压≤140/90mmHg；

⑹空腹血糖≤7.8mmol/L；

⑺糖化血红蛋白≤7.5% ；

⑻年龄25～75周岁，性别不限；

⑼自愿签署知情同意书者。

注：经过2周导入期后，需再次按照上述标准入选并随机入组。

## 4.5 排除标准

（1）原发性肾脏疾和其他可引起尿蛋白阳性的系统性疾病；

（2）各种其他内分泌代谢疾病；

（3）既往有心肌梗塞、心绞痛，既往3个月内有心脑血管事件；

（4）近期内（4周内）有各种感染者；

（5）妊娠或哺乳期妇女，对试验药物过敏者；

（6）既往3个月内用皮质激素、β受体阻滞剂、噻嗪类利尿剂及烟酸等药物治疗者；

（7）空腹血浆甘油三酯水平>l0mmol/L(＞886m/dL)者。转氨酶升高至正常范围上限(ULN) 2倍以上者；

（8）精神障碍患者，或依从性差者。

注：符合上述条件的任何一条，则排除本研究。

# 5 治疗方案

## 5.1 试验药物

- 糖肾方：规格为每袋装4g。由江苏省江阴天江药业有限公司根据糖肾方处方制成配方颗粒。
- 安慰剂：主要成分为乳糖、麦芽糊精及食用色素。规格为每袋装4g，由江苏省江阴天江药业有限公司生产。

## 5.2 药物的包装

将每个受试者用药量包装成6盒，每盒含1个月的用药120袋。

每袋包装标签：

| 糖肾方  （本方供临床研究用）  规格：每袋装4克  ◇功能主治：益气滋阴，活血通络。适用于糖尿病肾病患者。  ◇用法用量：开水冲服，每次2袋，每日2次。  生产批号：0606320  有效期至：2010年06月  中日友好医院监制  江阴天江药业有限公司生产 |
| --- |

## 5.3 药物分配及记录

按各医院分配的药物编码，各医院分发相应编码的药物。

每个研究单位指定一名试验用药管理员。研究者筛选合格受试者，签署知情同意后，由试验用药管理员根据受试者就诊先后顺序和药物编码从小到大顺序逐例发药，并登记在《临床试验用药使用记录表》；将剩余药物集中返还申办单位或按程序销毁。每次访视，研究者记录试验用药发放量、受试者服用量和归还量。

## 5.4 给药方法

**⑴基础治疗方案**

1. 低盐糖尿病饮食，合理控制热量摄入，合理运动；参照2006年美国糖尿病协会指南；
2. ACEI/ARB：选用ACEI类或ARB类药物中的一种，试验期间不可更换药物种类；
3. 降糖、降血脂：按照2006年ADA发布的指南合理选择用药。

**⑵试验组**

在上述基础治疗方案基础上，给予糖肾方颗粒。

糖肾方：每次2袋（8g），每日2次，早、晚服用，开水冲服。

**⑶对照组**

在上述基础治疗方案基础上，给予安慰剂。

安慰剂：每次2袋（8g），每日2次，早、晚服用，开水冲服。

**⑷给药周期**

给药观察期24周，每4周随访一次。

## 5.5 试验期间合并用药的规定

**⑴试验期间允许使用的药物**

进入临床研究后，受试者原则上要保持入组时使用的药物种类及剂量，但血糖、血脂及血压控制不理想时，可以按照上述基础治疗方案规定的用药原则，酌情加减药物及调整使用剂量。

调整基础治疗方案规定的药物及使用剂量，需要详细记录原因、药物种类和剂量。

如受试者同时患有其他疾病，必须服用的治疗药物和治疗方法可继续使用，应尽量保持用法用量不变。

**⑵试验期间不允许使用的药物**

不得加用对血糖有影响的中药和具有抗血小板、抗凝血和抗纤溶作用的中西药，对本研究观察疗效有影响的中西药物。

受试者应将试验期间合并使用的药物及治疗方法，详细报告医生，研究者必须在病例报告中记录并加以说明，包括疾病名称、药物、剂量、用法、使用时间等，以便总结时加以分析和报告。

## 5.6 药品清点

研究者在受试者用药后每次访视时，回收其剩余药品及包装。评价受试者的依从性，用药率（即实际服药量/应服药量）＜80 % 或 ＞120 % 为依从性差。研究者应在病例报告表上及时记录（附表二）。

## 5.7 药品保存与发放

试验用药品由试验单位统一保存、管理。试验药品附有相应编号的应急信件，应急信件保存在参加单位机构办公室。研究者按受试者就诊先后顺序依药物编号从小号顺序发放试验药物，不得选择药物，该药物编号在整个试验过程中保持不变。

## 5.8 受试者退出试验的条件及步骤

5.8.1 研究者决定的退出

受试者退出试验是指已经入选的受试者在试验过程中出现了不宜继续进行试验的情况下，研究者决定该病例退出其试验。

①使用受试药物过程中，原有病情加重（治疗后2～4周内血肌酐快速上升＞基线值的50%； eGFR下降基线值的50%以上），研究者认为已经不宜继续观察的，可让该受试者退出试验，接受其他有效治疗。已服用12周观察药物的病例按治疗无效处理。

②试验中，受试者依从性差，使用药物达不到规定量的80% 或超过规定量的120%。

5.8.2 受试者自行退出试验

根据知情同意书的规定，受试者有权中途退出试验，或受试者虽未明确提出退出试验，但不再接受用药及检测而失访，也属于“退出”。尽可能了解其退出的原因，并加以记录。如：自觉疗效不佳；对某些不良反应感到难以耐受；有事不能继续接受临床研究；经济因素；意外（如车祸）或未说明原因而失访等。

无论何种原因，对退出试验的病例，保留其病例记录表，对其疗效和不良反应进行分析。

## 5.9 剔除及脱落病例标准

5.9.1 脱落的判定

所有填写了知情同意书并筛选合格进入试验的受试者，均有权随时退出临床试验，无论何时何因退出，只要没有完成临床试验全程观察，均为脱落病例。

脱落病例的处理：当受试者脱落后，研究者必须在CRF中填写脱落原因，并尽可能与受试者联系，完成所能完成的评估项目，并填写治疗末次随访记录表，尽可能记录最后一次服药时间。对因不良事件而脱落，必须记录在CRF并通知申办者。

5.9.2 剔除病例标准

①病例选择违反了入组标准。

②未曾使用试验用药。

③在随机化之后没有任何数据。

# 6 有效性及安全性评价

**结局测量**

疗效指标的测量，每4周随访1次。

**主要疗效指标**

糖尿病肾病Ⅲ期以UAER为主要疗效指标，Ⅳ期的主要疗效指标为24小时尿蛋白定量。

**次要疗效指标**

1. 肾功能：血清肌酐、BUN、肾小球滤过率（采用Cockcroft-Grault公式估算）；
2. 血脂：总胆固醇、甘油三酯、高密度脂蛋白胆固醇、低密度脂蛋白胆固醇；
3. 中医症状评分：按症状无、轻、中、重分别评分；

4）生存质量评价：采用WHO QOL-BREF和糖尿病特异生存质量量表进行。

**安全性指标**

血、尿常规，心电图，ALT、AST。

# 7 不良事件监控

## 7.1 定义

不良事件（Adverse Event，AE）：不良事件的术语涵盖了在临床研究期间，受试者出现并会影响健康的任何临床症状、综合征或某种疾病出现或恶化。该术语也包括了实验室及在受试过程中发生与临床相关的其他情况，如需计划外诊治措施，或导致从试验中退出。不良事件可能是：新的疾病；治疗状态症状或体征的恶化，或伴随疾病的恶化；对照药物的作用；与参加该试验无关；一个或多个因素的组合。所以，“不良事件”这一术语并不意味着与试验药物的因果关系。

严重不良事件（Serious Adverse Event，SAE）：是在试验药物任何剂量下或在观察期间任何时候出现以下不良事件，包括：导致死亡；即刻危及受试者生命；需住院治疗或延长住院时间；伤残；导致先天畸形；有重要的医学意义（指那些不会立即危及生命或导致死亡或需住院的事件，但可能危害受试者或需要采取措施来预防上述所定义的一种后果）需要医学处理来防止永久性的伤害或损害。

药品不良反应（Adverse drug Reaction，ADR）：在按规定剂量正常应用药品的过程中产生的有害而非所期望的、与药品应用有因果关系的反应。在一种新药或药品新用途的临床试验中，其治疗剂量尚未确定时，一切有害而非所期望的、与药品应用有因果关系的反应，也应视为药品不良反应。

## 7.2严重程度的判断

不良事件的严重程度采用下列定义：

1.轻：患者较易忍受，或是引起轻微的不适，但不影响日常生活。

2.中：可引起病人不适，从而影响正常的日常生活。

3.重：患者丧失了正常生活的能力或阻碍了正常的日常生活。

## 7.3本研究预期可能发生的不良反应：

1. 糖肾方中含有大黄，有可能出现的不良反应包括：
2. **腹泻：**本方中的大黄具有“泻热毒，破积滞，行瘀血”的功效，服药后可能会出现大便呈半糊状或次数增多现象，如呈水样或每天大便3次以上需减量使用。
3. **结肠黑变病：**文献报道长期服用蒽醌类泻药会引起结肠黑病变。大黄的主要泻下成分为蒽醌类衍生物三羟甲基蒽醌。结肠黑变病表现为结肠黏膜表面有褐色素沉着，黏膜下层巨噬细胞胞质中含褐色质颗粒，诊断主要根据镜下检查和标本活检。结肠黑变病可能会增加大肠腺瘤的发病风险。
4. **其他不良反应：**有报道发现服用大黄可导致ALT和血肌酐升高，但多是由于服用过量或不遵医嘱用药所致，临床少见。

（二）ACEI/ARB的不良反应

干咳、肾功能恶化和高钾血症是ACEI/ARB类药物的最常见不良反应。具体内容见各药物的说明书。

## 8.4 与药物因果关系判断

## 8.4.1 不良反应判断的因果判断指标

根据我国《药品不良反应报告和监测管理办法》，使用的分析方法主要遵循以下五条原则。

⑴用药与不良反应/事件的出现有无合理的时间关系？ 有□ 无□

⑵反应是否符合该药已知的不良反应类型？ 是□ 否□ 不明□

⑶停药或减量后，反应/事件是否消失或减轻？ 是□ 否□ 不明□ 未停药或未减量□

⑷再次使用可疑药品后是否再次出现同样反应/事件？ 是□ 否□ 不明□ 未再使用□

⑸反应/事件是否可用并用药的作用、受试者病情的进展、其他治疗的影响来解释？是□否□不明□

## 8.4.2 因果判定标准

关联性评价：依据以上不良反应/事件分析的五条原则将关联性评价分为肯定、很可能、可能、可能无关、待评价、无法评价6级。

|  | 1 | 2 | 3 | 4 | 5 |
| --- | --- | --- | --- | --- | --- |
| 肯定 | ＋ | ＋ | ＋ | ＋ | － |
| 很可能 | ＋ | ＋ | ＋ | ？ | － |
| 可能 | ＋ | ± | ±？ | ？ | ±？ |
| 可能无关 | － | － | ±？ | ？ | ±？ |
| 待评价 | 需要补充材料才能评价 | | | | |
| 无法评价 | 评价的必须资料无法获得 | | | | |

注：＋表示肯定； －表示否定； ±表示难以肯定或否定； ？表示不明

## 8.5不良事件处理

## 8.5.1报告方法

发生任何不良事件，如受试者的主观不适及实验室检测异常，均需认真对待，仔细分析，立即采取措施保护受试者的安全。

## 8.5.2 处理程序

详细记录于CRF中，记录其持续、转归、消失等情况，及时对受试者进行复查及随访。

## 8.5.3 严重不良事件的处理

发生在试验过程中的任何严重不良事件，必须立即报告本单位和课题负责单位医学伦理委员会，填写“严重不良事件报告表”（附表三），按相应的程序进行报告。

## 8.5.4 随访未缓解的不良事件

所有不良事件都应当追踪，直到得到妥善解决或病情稳定。

# 9 数据管理

## 9.1 填写CRF

研究者根据受试者的原始观察记录，将及时、完整、正确、清晰地填写病例报告表。

## 9.2 核对CRF

监查员监督临床研究是否遵循研究方案。确认所有病例报告表填写正确完整，并与原始资料一致。如有错误和遗漏，及时要求研究者更正。修改时需保持原有记录清晰可见，改正处需经研究者签名并注明日期。

## 9.3 收回CRF

经过监查员检查的病例报告表，由监查员核查签字后，及时送交临床试验数据管理部门的管理员。对于完成的病例报告表在研究者、监查员、数据管理员之间的传送应有专门的记录，收到时应有相应的签名，记录需妥善保存。

## 9.4 发疑问表

数据管理员在数据录入前再次核查，发现问题及时通知监查员，要求研究者做出回答。他们之间的各种疑问及解答的交换应当采用疑问表形式，疑问表应保存备查。

## 9.5 建立数据库

数据管理员在进行数据录入前，要了解观察表格各项目的内容及编码情况，将编码工作过程记录于编码本保存。数据库命名应规范、易读、易查找。并保证其正确、安全和保密。

## 9.6 数据录入

数据录入员录入数据采用独立双份录入。录入过程发现问题或意外情况，应做好登记并及时报告，以便迅速处理问题，数据录入结束后应抽查部分观察表格，了解录入质量，分析并处理存在的问题。

## 9.7 数据库核查

数据管理员应与主要研究者一起，按病例报告表中各指标数值的范围和相互关系拟定数据范围检查和逻辑检查内容。并编写相应的计算机程序，在输入前控制错误数据输入，找出错误原因加以改正，所有错误内容及修改结果应有记录并妥善保存。

## 9.8 数据备份

原始病例报告表在按要求完成数据录入和核查后，按编号的顺序归档保存，并填有检索目录等，以备查考。电子数据文件包括数据库、检查程序、分析程序、分析结果、编码本和说明文件等，应分类保存，并有多个备份保存于不同磁盘或记录介质上，妥善保存，防止损坏。所有原始档案按我国《药物临床试验质量管理规范》的规定期限保存。

## 9.9 盲态审核与揭盲规定

盲态审核是指最后一个病历报告表输入数据库以后，直到第一次揭盲之前，对数据库数据进行的核对和评价。

当所有病例报告表经双份输入并核对无误后，由数据管理员写出数据库检查报告，其内容包括试验完成情况（含脱落受试者清单）、入选/排除标准检查、完整性检查、逻辑一致性检查、离群数据检查、时间窗检查、合并用药检查、不良事件检查等。

在盲态审核会议上，由课题负责人、研究者代表、监查员、数据管理员和生物统计专业人员对受试者签署知情同意书、试验过程盲态保持情况和试验过程的紧急揭盲情况等做出审核，并对数据库检查报告中提出的问题做出决议，并写出盲态审核报告。数据库同时将被锁定。

数据锁定后由保存盲底的工作人员进行第一次揭盲，此次揭盲列出每个受试者所属的处理组别(如A组或B组)而并不标明哪一个为试验组或安慰剂组，交由统计分析人员输入计算机，与数据文件进行链接后，进行统计分析。当统计分析结束后，写出统计分析报告。在总结会上进行第二次揭盲，此次揭盲分别标明A、B两组中哪一组为试验组或对照组。

# 10 统计分析

## 10.1 一般原则

所有的统计检验均采用双侧检验，P值小于或等于0.05将被认为所检验的差别有统计意义。

定量指标的描述将计算例数、均数、标准差、中位数、最小值、最大值。分类指标的描述用各类的例数及百分数。

## 10.2 统计分析人群

有效性分析人群选具有基线数据和至少一个治疗后的评估的受试者。安全分析人群为所进入随机化的受试者。

## 10.3 统计分析方法

### 10.3.1 入选及完成情况

列出完成病例情况及脱落原因，描述入组病例及安全性、有效性分析数据集的例数。

### 10.3.2 基线均衡性分析

基线定义为0天。

基础值的均衡性分析针对病例入组时基本人口学特征、生命体征、疗效相关指标等，以说明各组基础情况是否可比。

### 10.3.3 影响试验评价因素比较

**⑴合并用药情况分析**

根据是否使用合并用药列出例数及百分数。

治疗期间所有合并用药将根据《当代药品商品名与别名辞典》（中国药学会组织编写，化学工业出版社医学图书出版中心出版发行）分类进行编码，按照编码后的系统和标准名计算两组合并用药的例数、例次以及使用率，并列表详细描述合并用药的用法用量、使用时间等。

### 10.3.4 有效性分析

主要疗效指标UAER和24尿蛋白定量分析将使用线性混合效应模型，如果有必要，基线尿蛋白水平和/或HbA1C作为协变量。所有次要疗效指标将用线性混合效应模型分析。安全性评价以卡方检验比较两组之间的不良事件。

如果主要疗效指标和次要疗效指标是非正态分布的数据，且进行数据变换后仍然无法近似正态，表明混合效应模型不适用，将采用非参数检验。

### 10.3.5 安全性分析

**不良事件：**根据是否发生不良事件列出例数及百分数，采用卡方检验/Fisher精确概率法进行比较。

治疗期间所有不良事件将根据《世界卫生组织药品不良反应术语集》(WHOART)进行编码，按照编码后的系统和标准名计算各组不良事件的例数、例次以及发生率。

试验期间所有不良事件的种类、严重程度及与试验药物的关系等将列表描述。

**与安全性有关的实验室检查：**对实验室检查以治疗前后交叉表（根据临床意义判断）的形式列出所有完成的检查项目，并列出疗后异常的检查项目。

# 11 质量控制

所有参与本研究的中医医师要求具备主治医师或以上职称，具备较丰富的临床经验及临床研究经历，要有在规定期限内完成临床观察的时间和精力保障。在试验开始前，对所有研究人员（包括中医医师、实验药物管理员、结局评价者）进行关于标准化操作流程的培训，以加深研究人员对临床研究方案及其各项指标的理解和认识。并设立专人监管研究进程、监督研究过程及检查研究结果记录的可靠性。同时，我们还设立了不良事件监管系统，不良事件的具体类型、开始和结束时间、临床表现、治疗过程和转归均要求记录在案。不良事件与药物的关系按卫生部药品不良反应监测中心制订的肯定有关、可能有关、可能无关、肯定无关、无法评价5级进行评定。肯定有关和可能有关计为药物的不良反应。

# 12 伦理原则

## 12.1 伦理委员会审批试验方案

临床研究方案由课题负责人与各参加临床研究的医生共同商定，申报伦理委员会审批后实施。若本方案在临床研究实施过程中进行了修订，需再次申保报理委员会批准后实施。如发现涉及研究用药的重要新资料则必须将知情同意书作书面修改送伦理委员会批准后，再次取得受试者同意。

## 12.2 受益与风险

受试者可能从本项临床试验获得的受益包括，将提前获得有临床应用前景的、将来可能被SFDA正式批准上市的新药治疗；受试者还可获得该制剂的免费治疗和相关体检。

受试者参加本项临床试验可能面临的风险包括，ACEI/ARB类药物可能出现的不良反应，或被分配至安慰剂组的受试者，所服用的安慰剂没有有效成分。研究者有权根据自己的判断予以对症处理或终止该病例的临床试验；同时制定了合并用药规定以及终止与退出研究病例的标准，保护受试者健康与利益。

## 12.3 受试者的医疗和保护

各临床研究单位的医生负责受试者的医疗，做出与临床研究相关的医疗决定，保证受试者在研究期间出现不良事件时得到适当的治疗。

医生对研究中发生的严重不良事件，应采取必要的措施以保证受试者的安全和权益，并及时向相关管理部门报告，同时向参加临床研究的其他医院通报。

受试者在临床研究期间将免费获得研究药物，免费进行试验理化检查。如果发生与研究药物有关的不良事件，还将得到免费的医疗。治疗中如病情变化，受试者或家属应及时和医生联系，医生可根据自己的判断，决定如何治疗。

## 12.4受试者隐私的保护

只有参与临床研究人员和监查员才可能接触到受试者的个人医疗记录，他们将签署“研究者声明”或“保密承诺”中包括的保密内容。数据处理时将采用“数据匿名”的方式，省略可识别受试者个体身份的信息。

## 12.5 知情同意的过程

筛选合格的受试者，研究者必须说明有关临床研究的详细情况，包括研究目的、研究程序、可能的受益和风险、受试者的权利和义务等，使受试者充分理解并有充分的时间考虑、所提问题均得到满意答复后表示同意，并签署“知情同意书”后方能开始临床研究。每一例受试者签署知情同意书时医生要将自己的联系电话留给受试者，以便受试者在出现病情变化时能够随时找到医生。

## 12.6 伦理学要求

临床研究必须遵循赫尔辛基宣言和我国有关临床试验的法律、法规及规范进行。在试验开始之前，研究方案应由临床研究单位的伦理委员会审查批准后方可实施。

每一位受试者入选本研究前，研究医师有责任以书面文字形式，向其或其指定代表人完整、全面地介绍本研究的目的、程序和可能的风险，应让受试者知道他们有权随时退出本研究。入选前必须给每位受试者或其亲属一份书面知情同意书，研究医师有责任让每位受试者在进入研究之前获得知情同意，知情同意书应作为临床试验文档保留备查。

# 13 临床研究预期进度和完成日期

## 13.1 临床研究开始时间

拟定于2007年1月开始启动临床试验。

## 13.2 临床研究完成时间

拟定于2009年12月完成。

# 附表

## 附表一 临床试验研究流程图

| 研究阶段 | 导入期（周） | 观察期（周） | | | | | | |
| --- | --- | --- | --- | --- | --- | --- | --- | --- |
|  | -2 | 0 | 4 | 8 | 12 | 16 | 20 | 24 |
| 病史记录 | √ |  |  |  |  |  |  |  |
| 体格检查 | √ | √ | √ | √ | √ | √ | √ | √ |
| 血、尿常规 | √ | √ |  |  | √ |  |  | √ |
| 心电图 | √ | √ |  |  | √ |  |  | √ |
| 肝功能（ALT、AST） | √ | √ |  |  | √ |  |  | √ |
| 肾功能（Cr、BUN、eGFR） | √ | √ |  |  | √ |  |  | √ |
| 糖化血红蛋白 | √ | √ |  |  | √ |  |  | √ |
| 尿蛋白（UAER或24h-UP） | √ | √ |  |  | √ |  |  | √ |
| 血脂（TC、TG、LDL、VLDL） | √ | √ |  |  | √ |  |  | √ |
| 中医证候积分 |  | √ | √ | √ | √ | √ | √ | √ |
| 量表（WHO QOL-BREF、 DQOL） |  | √ | √ | √ | √ | √ | √ | √ |
| 药品发放记录 |  | √ | √ | √ | √ | √ | √ |  |
| 剩余药品回收记录 |  |  | √ | √ | √ | √ | √ | √ |
| 不良事件 |  | √ | √ | √ | √ | √ | √ | √ |

### 附表二 试验用药品使用记录表

| 中 心 号 | 药物编号 | 受试者  姓 名 | 发放日期 | 给药数量 | 剩余药品返还量 | 药品管理员  签 名 |
| --- | --- | --- | --- | --- | --- | --- |
|  |  |  |  |  |  |  |
|  |  |  |  |  |  |  |
|  |  |  |  |  |  |  |
|  |  |  |  |  |  |  |
|  |  |  |  |  |  |  |
|  |  |  |  |  |  |  |
|  |  |  |  |  |  |  |
|  |  |  |  |  |  |  |
|  |  |  |  |  |  |  |
|  |  |  |  |  |  |  |
|  |  |  |  |  |  |  |
|  |  |  |  |  |  |  |
|  |  |  |  |  |  |  |
|  |  |  |  |  |  |  |
|  |  |  |  |  |  |  |
|  |  |  |  |  |  |  |
|  |  |  |  |  |  |  |
|  |  |  |  |  |  |  |
|  |  |  |  |  |  |  |
|  |  |  |  |  |  |  |
|  |  |  |  |  |  |  |
|  |  |  |  |  |  |  |
|  |  |  |  |  |  |  |
|  |  |  |  |  |  |  |
|  |  |  |  |  |  |  |
|  |  |  |  |  |  |  |
|  |  |  |  |  |  |  |

### 附表三 严重不良事件报告表(SAE)

课题编号： 编号：

| 报告类型 | □首次报告 □随访报告 □总结报告 | | | | 报告时间： 年 月 日 | | |
| --- | --- | --- | --- | --- | --- | --- | --- |
| 医疗机构及专业名称 |  | | | | 电话 |  | |
| 申报单位名称 |  | | | | 电话 |  | |
| 试验用药品名称 | 中文名称： | | | | | | |
|  | 英文名称： | | | | | | |
| 药品类别 | □中药 □化学药 □新生物制品 □放射性药□进口药 □其它 | | | | | | 第 类 |
| 临床研究分期： | □ 导入期 □观察治疗期 □继续随访期  □生物等效性试验 □临床验证 | | | | | | 剂型： |
| 受试者情况 | 姓名： | 性别： | | 出生年月： | | | 民族： |
|  | 疾病诊断： | | | | | | |
| SAE情况 | □导致住院 □延长住院时间 □伤残 □功能障碍  □危及生命或死亡 □其它 | | | | | | |
| SAE发生时间： 年 月 日 | | | SAE 反应严重程度：□轻度 □中度 □重度 | | | | |
| 对试验用药采取的措施 | □继续用药 □减少剂量 □药物暂停后又恢复 □停用药物 | | | | | | |
| SAE转归 | □ 症状消失（后遗症 □有 □无） □症状持续  □ 死亡（死亡时间： 年 月 日） | | | | | | |
| SAE与试验药的关系 | □肯定有关 □可能有关 □可能无关 □无关 □无法判定 | | | | | | |
| SAE 报道情况 | 国内：□有 □无 □不祥 国外：□有 □无 □不祥 | | | | | | |
| SAE发生及处理的详细情况： | | | | | | | |

报告单位名称（盖章）： 报告人职务/职称： 报告人签名：

1. 特殊说明：“华北煤炭医学院”于2010年更名为“河北联合大学”，因此文章正文中参加单位写为“Hebei United University Affiliated Hospital”。 [↑](#footnote-ref-2)
2. 参考文献

   [?] Gu D, Reynolds K, Duan X, Xin X, Chen J, Wu X, et al. Prevalence of diabetes and impaired fasting glucose in the Chinese adult population: International Collaborative Study of Cardiovascular Disease in Asia (InterASIA) [J]. Diabetologia 2003; 46:1190-8 [↑](#endnote-ref-2)
3. [?] Dialysis and Transplantation Registration Group, Chinese Society of Nephrology. The report about the registration of dialysis and transplantation in China 1999[J]. Chin J Nephrol 2001; 17: 77-8. [↑](#endnote-ref-3)
4. [?] Ruggenenti P, Fassi A, Ilieva A P, Bruno S, Iliev I P, Bruseqan V, et al. Preventing microalbuminuria in type 2 diabetes[J]. N Engl J Med. 2004;351:1941-51. [↑](#endnote-ref-4)
5. [?] Barry MB, Mark EC, Dick DE Z, William FK, William EM, Hans-Henrik P, et al. Effects of Losartan on renal and cardiovascular out comes in patients with type 2 diabetes and nephropathy[J]. N Engl J Med. 2001; 345: 861-869. [↑](#endnote-ref-5)
6. [?] 张丽芬，赵进喜，吕仁和，杨洪涛，曹世丽，黄学民，等.糖尿病肾病肾功能不全防治优化方案的有效性和安全性研究[J].中医杂志. 2006; 47: 755-758. [↑](#endnote-ref-6)
7. [?] 赵进喜，牟新，王世东，宋美龄，黄学民，于秀辰，等。止消温肾宁颗粒剂治疗糖尿病肾病肾功能不全代偿期31例临床观察[J]. 中医杂志. 2005; 46:677-679. [↑](#endnote-ref-7)
8. [?] 宋美玲，杨敏，牟新，张丽芬，赵进喜. 中医辨证治疗糖尿病肾病肾功能不全的肾功能指标疗效和证候疗效评价[J]. 北京中医药大学学报.2006;29:429-432. [↑](#endnote-ref-8)
9. [?] 冯建春，倪青.糖肾胶囊治疗糖尿病肾病的临床研究[J].中国中西医结合杂志. 2000;20:212-214. [↑](#endnote-ref-9)
10. [?] American Diabetes Association. Standards of Medical Care in Diabetes: 2006[J]. Diabetes Care. 2006;29: 4-42. [↑](#endnote-ref-10)
11. [?]Mogensen C E, Christensen C K, Vittinghus E. The stages in diabetic renal disease: with emphasis on the stage of incipient diabetic nephropathy[J]. Diabetes, 1983, 32: 64-78. [↑](#endnote-ref-11)
12. [?] 郑筱萸.中药新药临床研究指导原则[M]. 北京：中国医药科技出版社；2002:163-168. [↑](#endnote-ref-12)
