# Supplementary material for: Efficacy and Safety of Tangshen Formula on Patients with Type 2 Diabetic Kidney Disease: A Multicenter Double-Blinded Randomized Placebo-Controlled Trial
Source: PLoS One. 2015 May 4;10(5):e0126027. doi: 10.1371/journal.pone.0126027 (PMC4418676; doi:10.1371/journal.pone.0126027)
Supplement: S1 Table — (DOC) [file pone.0126027.s005.doc]

**S1 Table.** **Chinese medicine symptomatology score survey instrument.**

| **Fatigue** | 🞎 0：None  🞎 2：Cannot sustain heavy work  🞎 4：Can manage mild intensity work  🞎 6：Can only do daily activities |
| --- | --- |
| **Weakness and soreness of the low back and knees** | 🞎 0：None  🞎 2：Occasionally weakness and soreness of the low back and knees  🞎 4：Weakness and soreness of the low back and knees,   need to alter body position for relief  🞎 6：Sustained pain of the low back and knees, need to take pain   medication for relief |
| **Heat sensation in the palms and soles** | 🞎 0：None  🞎 2：Occasional  🞎 4：Desire to expose extremities to the air  🞎 6：Desire to hold something cold |
| **Dry mouth and throat** | 🞎 0：None  🞎 2：Mild  🞎 4：Decreased saliva production  🞎 6：Severe thirst with constant need to drink fluids |
| **Qi deficiency and**  **listlessness** | 🞎 0：None  🞎 2：Shortness of breath after mild exercise  🞎 4：Shortness of breath after moderate exercise  🞎 6：Unable to talk or catch breath even without exercise |
| **Catch cold easily** | 🞎 0：None  🞎 1：Catch cold more than 6 times per year  🞎 2：Catch cold more than 10 times per year  🞎 3：Catch cold more than 12 times per year |
| **Pale complexion** | 🞎 0：None  🞎 1：Mild  🞎 2：Moderate  🞎 3：Pale or dark yellow complexion |
| **Irritability** | 🞎 0：None  🞎 1：Occasional  🞎 2：Easily irritable, but able to gain self-control  🞎 3：Severe agitation, unable to gain self-control |
| **Numbness** | 🞎 0：None  🞎 1：Hands and feet  🞎 2：Limbs  🞎 3：Entire body |
| **Edema** | 🞎 0：None  🞎 1：Palpebral edema in the morning  🞎 2：Palpebral and lower limb edema  🞎 3：Extreme generalized edema (hyposarca) |
| **Frequency of urination at night** | 🞎 0：None  🞎 1：2 times  🞎 2：3 to 4 times  🞎 3：5 times or more |
| **Constipation** | 🞎 0：None  🞎 1：Hard stools and exertion  🞎 2：Hard stools with a 2 to 3 day defecation interval  🞎 3：Hard stools with 3 or more days defecation interval |
| **Hematuria** | 🞎 0：None  🞎 1：Yes |
