# Supplementary material for: Efficacy and Safety of Tangshen Formula on Patients with Type 2 Diabetic Kidney Disease: A Multicenter Double-Blinded Randomized Placebo-Controlled Trial
Source: PLoS One. 2015 May 4;10(5):e0126027. doi: 10.1371/journal.pone.0126027 (PMC4418676; doi:10.1371/journal.pone.0126027)
Supplement: S2 Table — (DOC) [file pone.0126027.s006.doc]

**S2 Table. Nine most representative compounds in TSF identified by high-performance liquid chromatography/mass spectrometry.**

| **Peak Number** | **t_ms_ min** | **Representative Compound** | **Original Herbal Medicine** |
| --- | --- | --- | --- |
| 1 | 28.757 | Sweroside | *Cornus officinalis* Sieb. et Zuce |
| 2 | 39.052 | Rhapontigenin | *Rheum palmatum* L. |
| 3 | 42.164 | Isomucronulatol-7,2'-di-glucoside | *Astragalus membranaceus* (Fisch.) Bge. |
| 4 | 53.420 | Naringin | *Citrus aurantium* L. |
| 5 | 55.082 | Isonaringin | *Citrus aurantium* L. |
| 6 | 59.442 | Melittoside | *Rehmannia glutinosa* Libosch |
| 7 | 62.228 | Ginsenoside Rg1 | *Panax notoginseng* (Burk.) F.H. Chen |
| 8 | 74.350 | Morroniside | *Cornus officinalis* Sieb. et Zuce |
| 9 | 78.530 | Ginsenoside Rb1 | *Panax notoginseng* (Burk.) F.H. Chen |
