# Supplementary material for: Efficacy and Safety of Tangshen Formula on Patients with Type 2 Diabetic Kidney Disease: A Multicenter Double-Blinded Randomized Placebo-Controlled Trial
Source: PLoS One. 2015 May 4;10(5):e0126027. doi: 10.1371/journal.pone.0126027 (PMC4418676; doi:10.1371/journal.pone.0126027)
Supplement: S3 Table — (DOC) [file pone.0126027.s007.doc]

**S3 Table. WHOQOL-BREF scores in four domains and overall in microalbuminuria stage.**

| **Domains** | **Groups** | **Baseline** | **Week 12** | **Week 24** | **F** | ***P*** ^a^ |
| --- | --- | --- | --- | --- | --- | --- |
| Physical | SCM916 | 56.91±17.29 | 58.85±15.53 | 57.99±16.32 | 0.42 | 0.6557 |
|  | PLA | 65.98±18.30 | 66.49±16.88 | 64.45±15.09 |  |  |
| Psychological | SCM916 | 55.49±11.09 | 52.57±11.57 | 52.81±10.76 | 0.36 | 0.6980 |
|  | PLA | 59.12±9.18 | 57.34±9.49 | 55.55±11.73 |  |  |
| Social | SCM916 | 60.56±13.34 | 61.25±13.22 | 60.87±13.13 | 0.92 | 0.3993 |
|  | PLA | 63.66±15.93 | 67.00±15.30 | 65.27±13.52 |  |  |
| Environmental | SCM916 | 59.15±14.65 | 60.31±14.28 | 60.46±14.66 | 4.04 | 0.0199 |
|  | PLA | 66.12±17.51 | 65.62±16.06 | 59.37±14.01 |  |  |
| Overall QoL/health | SCM916 | 58.84±10.06 | 58.78±10.67 | 58.093±11.52 | 0.70 | 0.4980 |
|  | PLA | 64.55±13.86 | 64.45±12.09 | 61.16±10.54 |  |  |

^a^ MANOVA of repeated measuring, *P*<0.05 was considered significant.
PLA = placebo.
