# Supplementary material for: Efficacy and Safety of Tangshen Formula on Patients with Type 2 Diabetic Kidney Disease: A Multicenter Double-Blinded Randomized Placebo-Controlled Trial
Source: PLoS One. 2015 May 4;10(5):e0126027. doi: 10.1371/journal.pone.0126027 (PMC4418676; doi:10.1371/journal.pone.0126027)
Supplement: S4 Table — (DOC) [file pone.0126027.s008.doc]

**S4 Table. WHOQOL-BREF scores in four domains and overall in macroalbuminuria stage.**

| **Domains** | **Groups** | **Baseline** | **Week 12** | **Week 24** | **F** | ***P* ^a^** |
| --- | --- | --- | --- | --- | --- | --- |
| Physical | SCM916 | 65.85±14.71 | 62.12±15.04 | 63.20±14.10 | 1.42 | 0.2460 |
|  | PLA | 58.82±13.60 | 59.87±14.14 | 60.92±10.59 |  |  |
| Psychological | SCM916 | 54.78±12.48 | 55.30±11.03 | 54.16±9.88 | 0.80 | 0.4528 |
|  | PLA | 56.37±10.11 | 54.90±8.98 | 57.35±8.65 |  |  |
| Social | SCM916 | 64.58±12.52 | 64.32±12.20 | 66.16±12.31 | 1.10 | 0.3384 |
|  | PLA | 61.76±12.17 | 66.17±12.31 | 66.17±10.81 |  |  |
| Environmental | SCM916 | 66.44±13.15 | 64.10±11.93 | 62.12±12.49 | 4.08 | 0.0200 |
|  | PLA | 62.50±14.93 | 62.34±17.25 | 65.25±14.48 |  |  |
| Overall QoL/health | SCM916 | 63.32±11.02 | 60.55±13.16 | 61.41±10.25 | 1.39 | 0.2535 |
|  | PLA | 61.13±9.97 | 61.66±10.50 | 62.43±7.69 |  |  |

^a^MANOVA of repeated measuring, *P*<0.05 was considered significant.
PLA = placebo.
