# Supplementary material for: Efficacy and Safety of Tangshen Formula on Patients with Type 2 Diabetic Kidney Disease: A Multicenter Double-Blinded Randomized Placebo-Controlled Trial
Source: PLoS One. 2015 May 4;10(5):e0126027. doi: 10.1371/journal.pone.0126027 (PMC4418676; doi:10.1371/journal.pone.0126027)
Supplement: S5 Table — (DOC) [file pone.0126027.s009.doc]

**S5 Table. DQOL scores in four domains and overall in microalbuminuria stage.**

| **Domains** | **Groups** | **Baseline** | **Week 12** | **Week 24** | **F** | ***P^a^*** |
| --- | --- | --- | --- | --- | --- | --- |
| Physical | SCM916 | 27.01±6.73 | 26.65±6.45 | 26.74±6.28 | 0.54 | 0.5827 |
|  | PLA | 23.50±4.45 | 22.87±4.75 | 23.57±5.14 |  |  |
| Psychological | SCM916 | 19.50±4.55 | 19.20±5.38 | 20.34±4.37 | 0.89 | 0.4147 |
|  | PLA | 18.48±4.78 | 15.66±7.06 | 18.76±4.06 |  |  |
| Social | SCM916 | 7.07±2.34 | 7.52±2.55 | 6.85±2.76 | 0.11 | 0.8969 |
|  | PLA | 6.65±1.98 | 7.20±2.28 | 6.95±2.14 |  |  |
| Treatment | SCM916 | 5.88±1.27 | 5.83±1.28 | 5.83±1.30 | 0.30 | 0.7391 |
|  | PLA | 5.70±1.08 | 5.75±1.12 | 5.85±1.18 |  |  |
| Overall DSQL scores | SCM916 | 59.31±12.35 | 59.07±12.31 | 60.26±12.20 | 0.33 | 0.7230 |
|  | PLA | 53.79±9.90 | 53.08±11.06 | 55.33±9.86 |  |  |

^a^MANOVA of repeated measuring, *P*<0.05 was considered significant.
PLA = placebo.
