# Supplementary material for: Efficacy and Safety of Tangshen Formula on Patients with Type 2 Diabetic Kidney Disease: A Multicenter Double-Blinded Randomized Placebo-Controlled Trial
Source: PLoS One. 2015 May 4;10(5):e0126027. doi: 10.1371/journal.pone.0126027 (PMC4418676; doi:10.1371/journal.pone.0126027)
Supplement: S6 Table — (DOC) [file pone.0126027.s010.doc]

**S6 Table. DQOL scores in the four domains and overall in macroalbuminuria stage.**

| **Domains** | **Groups** | **Baseline** | **Week 12** | **Week 24** | **F** | ***P*^a^** |
| --- | --- | --- | --- | --- | --- | --- |
| Physical | SCM916 | 27.51±7.92 | 26.46±8.24 | 27.39±7.73 | 0.23 | 0.7933 |
|  | PLA | 26.45±7.24 | 26.45±7.88 | 27.41±7.78 |  |  |
| Psychological | SCM916 | 19.93±5.51 | 18.57±6.81 | 19.18±5.59 | 0.70 | 0.4971 |
|  | PLA | 18.30±6.59 | 18.09±6.85 | 19.88±5.15 |  |  |
| Social | SCM916 | 6.64±1.62 | 7.00±1.93 | 7.16±2.45 | 0.57 | 0.5661 |
|  | PLA | 7.55±2.74 | 7.33±2.42 | 7.26±2.14 |  |  |
| Treatment | SCM916 | 5.81±1.58 | 5.81±1.48 | 6.02±1.83 | 0.23 | 0.7957 |
|  | PLA | 5.50±1.46 | 5.55±1.46 | 5.94±1.86 |  |  |
| Overall DSQL scores | SCM916 | 59.74±13.54 | 57.97±15.12 | 59.91±15.35 | 0.21 | 0.8080 |
|  | PLA | 58.25±14.70 | 57.90±15.49 | 60.70±14.50 |  |  |

MANOVA of repeated measuring, *P*<0.05 was considered significant.
PLA = placebo.
